# Delineation of phenotypes and genotypes related to cohesin structural protein RAD21

**DOI:** 10.1007/s00439-020-02138-2

**Published:** 2020-03-19

**Authors:** Lianne C. Krab, Iñigo Marcos-Alcalde, Melissa Assaf, Meena Balasubramanian, Janne Bayer Andersen, Anne-Marie Bisgaard, David R. Fitzpatrick, Sanna Gudmundsson, Sylvia A. Huisman, Tugba Kalayci, Saskia M. Maas, Francisco Martinez, Shane McKee, Leonie A. Menke, Paul A. Mulder, Oliver D. Murch, Michael Parker, Juan Pie, Feliciano J. Ramos, Claudine Rieubland, Jill A. Rosenfeld Mokry, Emanuela Scarano, Marwan Shinawi, Paulino Gómez-Puertas, Zeynep Tümer, Raoul C. Hennekam

**Affiliations:** 1 Department of Pediatrics, Amsterdam UMC, University of Amsterdam, Meibergdreef 9, 1105AZ Amsterdam, The Netherlands; 2 Cordaan, Outpatient Clinic for ID Medicine, Klinkerweg 75, 1033 PK Amsterdam, The Netherlands; 3 Odion, Outpatient Clinic for ID Medicine, Purmerend, The Netherlands; 4 Molecular Modelling Group, Centro de Biología Molecular Severo Ochoa, CBMSO (CSIC-UAM), Madrid, Spain; 5 School of Experimental Sciences-IIB, Universidad Francisco de Vitoria, UFV, Pozuelo de Alarcón, Spain; 6 Banner Childrens Specialists Neurology Clinic, Glendale, AZ USA; 7 Clinical Genetics Service, Sheffield Children’s Hospital, Academic Unit for Child Health, University of Sheffield, Sheffield, UK; 8 Department of Clinical Genetics, Kennedy Center, Copenhagen University Hospital, Rigshospitalet, Gl. Landevej 7, 2600 Glostrup, Denmark; 9 Department of Pediatrics and Adolescent Medicine, Copenhagen University Hospital, Rigshospitalet, Glostrup, Denmark; 10 MRC Human Genetics Unit, University of Edinburgh, Edinburgh, UK; 11 Department of Immunology, Genetics and Pathology, Uppsala University, Uppsala, Sweden; 12 Prinsenstichting, Purmerend, The Netherlands; 13 Division of Medical Genetics, Department of Internal Medicine, Istanbul University, Istanbul, Turkey; 14 Department of Clinical Genetics, Amsterdam UMC, University of Amsterdam, Amsterdam, The Netherlands; 15 Unidad de Genética, Hospital Universitario y Politécnico La Fe, Valencia, Spain; 16 Northern Ireland Regional Genetics Service, Belfast City Hospital, Belfast, UK; 17 Autism Team Northern-Netherlands, Jonx Department of Youth Mental Health and Autism, Lentis Psychiatric Institute, Groningen, The Netherlands; 18 Institute of Medical Genetics, University Hospital of Wales, Cardiff, UK; 19 Clinical Genetic Service, Northern General Hospital, Sheffield, UK; 20 Unit of Clinical Genetics Unit, Service of Pediatrics, University Hospital “Lozano Blesa”, University of Zaragoza School of Medicine, Saragossa, Spain; 21 Unit of Clinical Genetics Unit and Functional Genomics, Department of Pharmacology and Physiology, University of Zaragoza School of Medicine, Saragossa, Spain; 22 Department of Pediatrics, Division of Human Genetics, Inselspital, University of Bern, Bern, Switzerland; 23 Department of Molecular and Human Genetics, Baylor College of Medicine, Baylor Genetics Laboratories, Houston, TX USA; 24 Rare Disease Unit, Department of Pediatrics, St. Orsola Hospital, Bologna, Italy; 25 Department of Pediatrics, Division of Genetics and Genomic Medicine, Washington University School of Medicine, St. Louis, MO USA; 26 Department of Clinical Medicine, University of Copenhagen, Copenhagen, Denmark

## Abstract

*RAD21* encodes a key component of the cohesin complex, and variants in *RAD21* have been associated with Cornelia de Lange Syndrome (CdLS). Limited information on phenotypes attributable to *RAD21* variants and genotype–phenotype relationships is currently published. We gathered a series of 49 individuals from 33 families with *RAD21* alterations [24 different intragenic sequence variants (2 recurrent), 7 unique microdeletions], including 24 hitherto unpublished cases. We evaluated consequences of 12 intragenic variants by protein modelling and molecular dynamic studies. Full clinical information was available for 29 individuals. Their phenotype is an attenuated CdLS phenotype compared to that caused by variants in *NIPBL* or *SMC1A* for facial morphology, limb anomalies, and especially for cognition and behavior. In the 20 individuals with limited clinical information, additional phenotypes include Mungan syndrome (in patients with biallelic variants) and holoprosencephaly, with or without CdLS characteristics. We describe several additional cases with phenotypes including sclerocornea, in which involvement of the *RAD21* variant is uncertain. Variants were frequently familial, and genotype–phenotype analyses demonstrated striking interfamilial and intrafamilial variability. Careful phenotyping is essential in interpreting consequences of *RAD21* variants, and protein modeling and dynamics can be helpful in determining pathogenicity. The current study should be helpful when counseling families with a *RAD21* variation.

**Electronic supplementary material:**

The online version of this article (10.1007/s00439-020-02138-2) contains supplementary material, which is available to authorized users.

## Introduction

**Fig. 1:**
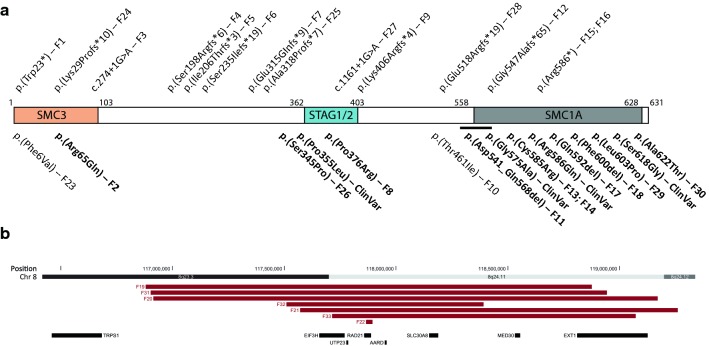
Presently reported *RAD21* variants. **a** RAD21 (horizontal bar) has three binding domains: SMC3 (p.1–103), STAG1/2 (p.362–403) and SMC1A (p.558–628). Sizes of the binding domains are not shown to scale. Truncating *RAD21* variants are shown above, and missense mutations and in-frame deletions are shown below the protein representation. Variants for which protein modelling is available, are marked in bold. *F* family number. The horizontal black line represents the inframe deletion p.(Asp541_Gln568del). *ClinVar* variants which are reported in the ClinVar database and could be investigated for pathogeneity with protein modelling (see supplementary Table S6). **b** Genomic region showing the microdeletions including *RAD21*

RAD21 (ENSG00000164754; OMIM *606462) is a key component of the cohesin complex and it forms a tri-partite ring together with SMC1A and SMC3 (Fig. 1 and Suppl. Fig. S1). The cohesin complex is a major modulator of chromosome structure, is involved in regulating chromosome segregation during mitosis, DNA repair and chromatin condensation, and plays an important role in gene transcription during interphase and cellular homeostasis (Kamada and Barilla 2018; Mullenders et al. 2015; Watrin et al. 2016). RAD21 has been implicated in additional processes including mediation of epigenetic silencing and induction of apoptosis (Fisher et al. 2017; Pati et al. 2002). Variants in genes encoding various structural or functional components of the cohesin complex, including *RAD21*, *SMC1A, SMC3, BRD4, STAG1/2, NIPBL, HDAC8, WAPL, ANKRD11* and in single individuals *PDS5A* and *ESPL1*, have been implicated in Cornelia de Lange Syndrome (CdLS) (Ansari et al. 2014; Kline et al. 2018; Woods et al. 2014; Yuan et al. 2019). *RAD21* spans ~ 29 Kb and has 14 exons (13 coding, 1 noncoding) that together encode a protein of 631 amino acids (McKay et al. 1996).

*RAD21* variants are found in a minority of CdLS patients. To date, nine missense variants and 5 microdeletions have been reported in CdLS patients (Kline et al. 2018). CdLS is characterized by distinct facial features, growth delay, microcephaly, limb reduction defects, intellectual disability (ID) and behavioral problems, especially self-injurious behavior (SIB) and autism spectrum disorder (ASD) (Kline et al. 2018). *RAD21* variants have also been associated with sclerocornea (Zhang et al. 2019) and Mungan syndrome (Chronic Idiopatic Intestinal Pseudoobstruction; OMIM #611376, in patients with biallelic *RAD21* variants) (Bonora et al. 2015; Mungan et al. 2003), each in a single family in which no remarks on CdLS features were made in the report. Loss of function-variants in cohesin genes including *RAD21* were found in individuals with holoprosencephaly of whom some demonstrated CdLS features as well (Kruszka et al. 2019).

*RAD21* is positioned on chromosome 8q24.11, between *TRPS1* (Tricho-Rhino-Phalangeal syndrome type 1; OMIM *604386) and *EXT1* (Multiple Exostoses type 1; OMIM *608177). Several microdeletions involving *RAD21* encompass genes next to *RAD21* (contiguous gene syndrome), complicating attribution to *RAD21* of the phenotype (Deardorff et al. 2012; Pereza et al. 2012; Wuyts et al. 2002). TRPS type 2 or Langer-Giedion syndrome (OMIM #150230) involves *TPRS1, RAD21* and *EXT1*, and the facial phenotype is mainly determined by loss of *TRPS1*, whereas the bony abnormalities arise from the loss of *EXT1* (Maas et al. 2015).

Based on the small case series of CdLS patients with *RAD21* variants reported so far, face and limb manifestations of CdLS seem to be less pronounced compared to individuals with variants in the other cohesin complex genes, and the impact on cognitive functioning seems attenuated, without clear genotype–phenotype correlation (Kline et al. 2018; Minor et al. 2014). Here, we report on a case series of 49 patients from 33 families with *RAD21* alterations, including all previously published cases with sequence variants, most of which with updated clinical data. We included 24 hitherto unpublished cases. We present genotype data, evaluate the pathogenicity of intragenic variants by a combination of phenotype, protein modelling, and molecular dynamic studies, and provide information on clinical phenotype, including cognitive and behavioral functioning, interfamilial and intrafamilial variability, and genotype–phenotype associations. We compare the *RAD21* phenotype to that of patients with *NIPBL* and *SMC1A* variants.

## Results

We identified 219 cases with *RAD21* variants, of which 49 patients from 33 families were included in this study (Tables 1 and S1). We describe in Table S6 those excluded cases that still may be of interest such as published cases with involvement of other morbid genes (Deardorff et al. 2012; Maas et al. 2015; Pereza et al. 2012; Wuyts et al. 2002; Yuen et al. 2015), variants reported as variant of unknown significance (VUS) that remained with unknown significance subsequent to re-evaluation, and cases for whom the relationship between phenotype and *RAD21* variant could not be confirmed(Kruszka et al. 2019; Zhang et al. 2019).

The 49 patients can be divided into two groups: cohort A includes 29 patients (22 families) with sufficient clinical data; and cohort B includes 20 patients (11 families) with incomplete data. Of the 49 cases, 24 are new. Twenty-five were previously published (Ansari et al. 2014; Bonora et al. 2015; Boyle et al. 2017; Deardorff et al. 2012; Dorval et al. 2019; Gudmundsson et al. 2018; Kruszka et al. 2019; Lee et al. 2014; Martinez et al. 2017; McBrien et al. 2008; Minor et al. 2014; Yuan et al. 2019), and for 19 of these clinical data could be updated (Table 1). Patients originated from Australia, Belgium, Canada, Denmark, Germany, Italy, Netherlands, Spain, Sweden, Switzerland, Turkey, United Kingdom and United States.

**Table 1:** Molecular findings of the presently reported series of individuals with *RAD21* variants

PID	Reference	Source	CdLS score^a^	Exon/intron	Nucleotide change	Predicted amino acid change	Type	Inheritance
Cohort A—sufficient clinical data								
F1	Martinez 2017	Updated	9	Exon 2	c.68G > A	p.(Trp23*)	Nonsense	De novo
F2	Clinvar	New	≥ 7	Exon 2	c.194G > A	p.(Arg65Gln)	Missense^b^	
F3a	Ansari 2014 P1	Updated	≥ 10	Intron 3	c.274 + 1G > A		Splice site	Familial (paternal)
F4	Minor 2014 P2	Updated	12	Exon 6	c.592_593dupAG	p.(Ser198Argfs*6)	Frameshift	
F5	Unpublished	New	9	Exon 6	c.617_620del	p.(Ile206Thrfs*3)	Frameshift	De novo
F6a	Boyle 2017 IV.16	Updated	12	Exon 7	c.704delG	p.(Ser235Ilefs*19)	Frameshift	Familial (maternal)
F6b	Boyle 2017 III.1	Updated	10	Exon 7	c.704delG	p.(Ser235Ilefs*19)	Frameshift	Familial (parents not tested)
F6c	Boyle 2017 III.2	Updated	9	Exon 7	c.704delG	p.(Ser235Ilefs*19)	Frameshift	Familial (parents not tested)
F6d	Boyle 2017 III.5	Updated	9	Exon 7	c.704delG	p.(Ser235Ilefs*19)	Frameshift	Familial (parents not tested)
F6e	Unpublished	New	12	Exon 7	c.704delG	p.(Ser235Ilefs*19)	Frameshift	Familial (maternal)
F7	Dorval 2019	Original data	≥ 11	Exon 9	c.943_946del	p.(Glu315Glnfs*9)	Frameshift	De novo
F8	Deardorff 2012 P5	Original data	≥ 10	Exon 9	c.1127C > G	p.(Pro376Arg)	Missense^b^	De novo
F9	Kruszka 2019 P14	Updated	13	Exon 10	c.1217_1224del	p.(Lys406Argfs*4)	Frameshift	De novo
F10	Unpublished	New	10	Exon 11	c.1382C > T	p.(Thr461Ile)	Missense	Familial (paternal)
F11a	Minor 2014 P1	Updated	8	Exon 13	c.1621-388_1704 + 193del	p.(Asp541_Gln568del)	Inframe deletion	Familial (maternal)
F11b	Minor 2014 mother P1	Updated	≥ 5	Exon 13	c.1621-388_1704 + 193del	p.(Asp541_Gln568del)	665 bp inframe deletion	
F12	Unpublished	New	13	Exon 13	c.1635del	p.(Gly547Alafs*65)	Frameshift	De novo
F13	Deardorff 2012, P6	Orginal data	≥ 12	Exon 14	c.1753T > C	p.(Cys585Arg)	Missense^b^	De novo
F14a	Unpublished	New	12	Exon 14	c.1753T > C	p.(Cys585Arg)	Missense^b^	Familial (parents not tested)
F14b	Unpublished	New	≥ 10	Exon 14	c.1753T > C	p.(Cys585Arg)	Missense	Familial (parents not tested)
F15	Unpublished	New	≥ 12	Exon 14	c.1756C > T	p.(Arg586*)	Nonsense	
F16a	Unpublished	New	10	Exon 14	c.1756C > T	p.(Arg586*)	Nonsense	Familial (paternal)
F16b	Father, unpublished	New	≥ 10	Exon 14	c.1756C > T	p.(Arg586*)	Nonsense	
F17	Gudmunsson 2019	Updated	8	Exon 14	c.1774_1776del	p.(Gln592del)	Inframe deletion^b^	De novo
F18	Unpublished	New	9	Exon 14	c.1800_1802del	p.(Phe600del)	Inframe deletion^b^	
F19	Deardorff 2012 P4	Original data	≥ 12	Whole gene	arr[hg19] 8q23.3q24.11(116880827–118875305)x1		2 Mb deletion	
F20	Unpublished	New	≥ 12	Whole gene	arr[hg19] 8q23.3q24.11(116915114–119171074)x1		2.3 Mb deletion	De novo
F21	Deardorff 2012 P2, McBrein 2008	Original data	≥ 12	Whole gene	arr[hg19] 8q23.3q24.12(117571728–119260904)x1		1.7 Mb deletion	De novo
F22	Unpublished	New	12	Exons 1–9	arr[hg19] 8q24.11(117866471–117893495)x1		27 kb deletion	
Cohort B—insufficient clinical data								
F3b	Ansari 2014	Updated		Intron 3	c.274 + 1G > A	n/a	Splice site	
F23	Decipher 271431	New		Exon 2	c.16T > G	p.(Phe6Val)	Missense	De novo
F24	Unpublished	New		Exon 2	c.85delinsCCT	p.(Lys29Profs*10)	Frameshift	
F25a	Decipher 272901	New		Exon 9	c.951del	p.(Ala318Profs*7)	Frameshift	Familial (paternal)
F25b	Decipher 272901 father	New		Exon 9	c.951del	p.(Ala318Profs*7)	Frameshift	
F26	Decipher 275402	New		Exon 9	c.1033T > C	p.(Ser345Pro)	Missense^b^	De novo
F27a	Yuan 2018 P2	Updated		Intron 10	c.1161 + 1G > A		Splice site	Familial (maternal)
F27b	Yuan 2018 mother P2	Updated		Intron 10	c.1161 + 1G > A		Splice site	
F28a	Kruszka 2019 P12/Yuan 2019 P1	Updated		Exon 12	c.1550dupC	p.(Glu518Argfs*19)	Frameshift	Familial (paternal)
F28b	Kruszka 2019 P12 father/Yuan 2019 P1 father	Updated		Exon 12	c.1550dupC	p.(Glu518Argfs*19)	Frameshift	
F29	Lee 2014 P76	Original data		Exon 14	c.1808T > C	p.(Leu603Pro)	Missense^b^	De novo
F30a	Bonora 2015 IV.9	Updated		Exon 14	c.[1864G > A];[1864G > A]	p.(Ala622Thr)	Missense^b^	Familial (both parents)
F30b	Bonora 2015 IV.10	Updated		Exon 14	c.[1864G > A];[1864G > A]	p.(Ala622Thr)	Missense^b^	Familial (both parents)
F30c	Bonora 2015 IV.11	Updated		Exon 14	c.[1864G > A];[1864G > A]	p.(Ala622Thr)	Missense^b^	Familial (both parents)
F30d	Unpublished	New		Exon 14	c.[1864G > A]	p.(Ala622Thr)	Missense^b^	Familial (nos)
F30e	Unpublished	New		Exon 14	c.[1864G > A]	p.(Ala622Thr)	Missense^b^	Familial (nos)
F30f	Unpublished	New		Exon 14	c.[1864G > A]	p.(Ala622Thr)	Missense^b^	Familial (nos)
F31	ClinVar	New		Whole gene	arr[hg19] 8q23.3-24.11(116902507–118942698)x1		2 Mb deletion; includes several genes	
F32	ClinVar	New		Whole gene	arr[hg19] 8q23.3-24.11(117509968–118391406)x1		880 kb deletion; includes several genes	
F33	ClinVar	New		Whole gene	arr[hg19] 8q24.11(117714768–119072307)x1		1.4 Mb deletion; includes several genes	

Cohort A: detailed clinical data available, including information on all cardinal CdLS features; cohort B: insufficient clinical data available

*F* family number, *P* patient number in the respective publication, *nos* not otherwise specified

^a^Based on (Kline et al. 2018); ≥ defines at least (minor criteria missing). Score < 4 is insufficient to indicate molecular testing for CdLS; score 4–8 indicates molecular testing for CdLS indicated; score 9–10 indicates non-classic CdLS; score 11 or higher indicates classic CdLS

^b^Variants investigated with protein modelling

### Genotype

The 33 families harbor 31 different variants: seven unique copy number variations (CNVs) and 24 intragenic sequence variants. Two of the latter were recurrent [p.(Cys585Arg) and p.(Arg586*), each found in 2 families (Table 1, Fig. 1)]. A relatively large proportion of the cases are familial (nine out of 21 index cases for whom inheritance could be established). The seven CNVs were all deletions, six of which included other genes in addition to *RAD21*. Of the 24 different sequence variants, 13 are predicted be truncating (2 nonsense, 2 splice site and 9 frameshift variants), and these are scattered throughout the gene. Three of the variants are in-frame deletions, two of which affect a single amino acid, while the 665 bp deletion includes the whole exon 13. The missense variants tend to cluster at the functional domains of the protein. Some variants in cohort B may be recurrent but sufficient data are lacking to confirm this (Table S6).

### Evaluation of pathogenicity of *RAD21* variants using molecular dynamic analyses

**Fig. 2:**
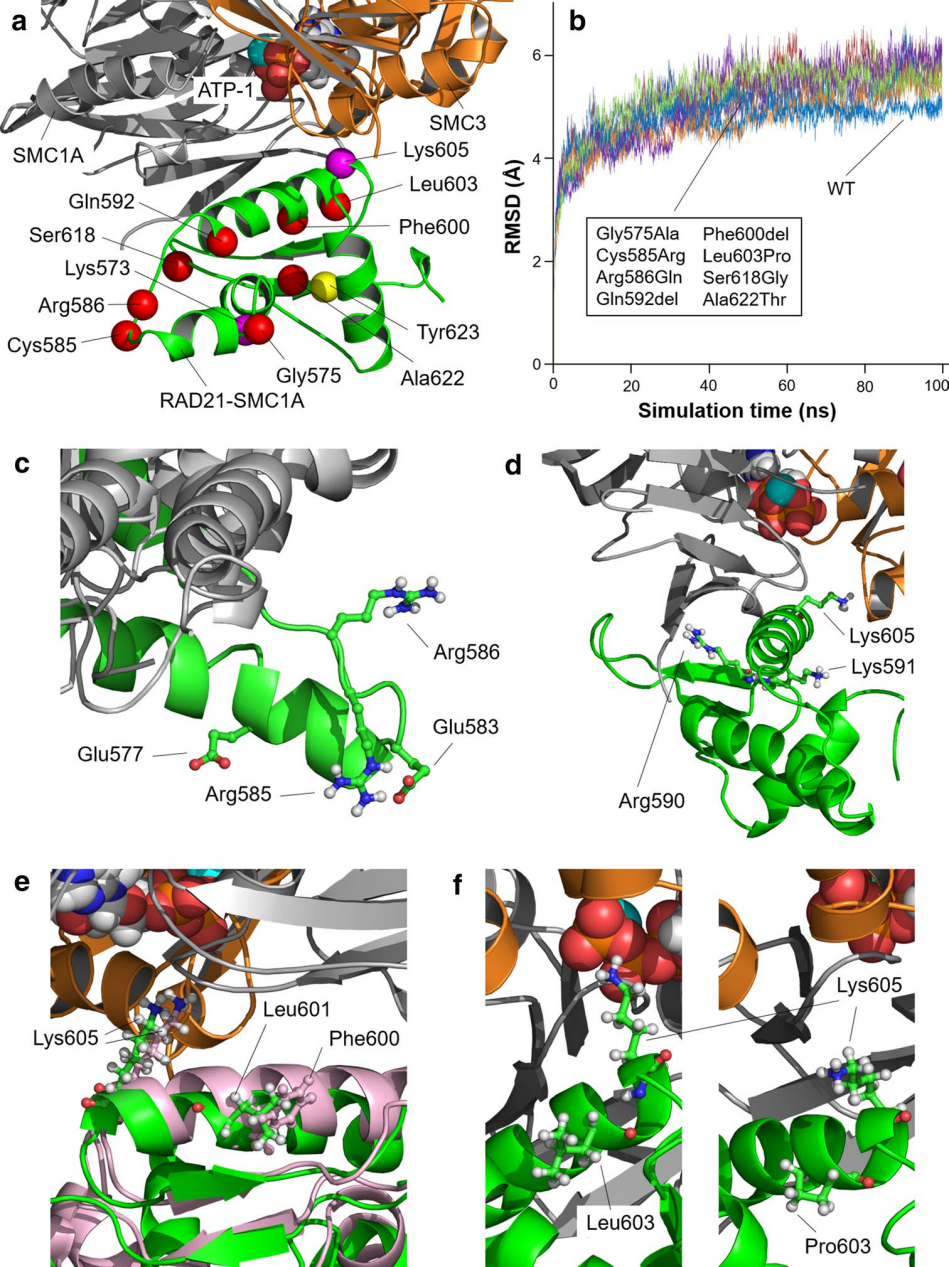
Structural modeling of RAD21-SMC1A domain bound to the head domain of SMC1A/SMC3 complex. **a** Model for the RAD21-SMC1A domain (residues 543–628, green) associated to the head domains of SMC1A (grey) and SMC3 (orange), close to the ATP molecule in ATPase site 1 (ATP-1) of the SMC1A/SMC3 dimer. Position of affected residues (Gly575, Cys585, Arg586, Gln592, Phe600, Leu603, Ser618 and Ala622) is indicated as red spheres. Locations of other important residues (Lys573, Gly575, Lys605, and Thr623) are indicated. Residue Cys585 is located next to residue Arg586. Residue Arg586 interacts through a salt bridge with RAD21 residue Glu577, stabilizing RAD21-SMC1A structure. Three mutated residues (Gln592, Phe600, Leu603) are located in the same alpha-helix as key residue Lys605, predicted to maintain the correct positioning of SMC1A-Asn35 at ATPase site 1, putting it into contact with a catalytic water molecule and, thus, allowing progression of the ATPase reaction, pivotal to opening of the cohesin ring and the cyclic process (Marcos-Alcalde et al. 2017). Variants Ser618Gly and Ala622Thr do not cause structural alterations. **b** Root mean square deviation (RMSD, in Angstroms) of modeled structures (WT, wild-type, blue line; Gly575Ala, red; Cys585Arg, light purple; Arg586Gln, dark green; Gln592del, light blue; Phe600del, orange; Leu603Pro, cyan; Ser618Gly, dark purple; Ala622Thr, light green. No relevant differences in RMSD values demonstrable in the trajectories of the mutated models when compared with wild-type model and with one another. **c** Variant Cys585Arg causes the adjacent Arg586 to lose interaction with Glu577, and both the Arg586 and Glu577 residues change their position in the mutant protein by pointing towards the solvent, which modifies the distribution of charges in the surface of RAD21-SMC1A, while the new Arg585 residue is stabilized in a novel interaction with Glu583. **d** Model for variant Gln592del after 100 ns of MD. New positions of Arg590, Lys591 and Lys605 due to the absence of Gln592 are indicated. Deletion of Gln592 residue causes adjacent Lys591 to be located in the same position as the missing amino acid. This situation promotes a conformational change in the alpha helix, causing the Lys605, which is placed in the same alpha helix, to move away from site 1 of the ATPase. **e** Model for variant Phe600del (green) compared to wild-type model (pink) after 100 ns of MD. Despite the local rearrangement in the alpha helix, distortions of the alpha helix are not relevant as residue Leu601 is placed spatially in the position equivalent to the deleted Phe600 during the MD trajectory, allowing Lys605 to remain in the same position. **f** Structure of wild-type RAD21-SMC1A (left) and variant Leu603Pro (right) models after 100 ns of MD. Presence of mutated Pro603 instead of wild-type Leu603 promotes a local change in the bending angle of the alpha-helix in which it is located, resulting in a conformational change in the alpha helix that moves Lys605 out of its initial position close to ATPase site 1

For 12 intragenic variants (ten missense variants and two 3 bp in-frame deletions, from individuals in cohort A, B and Table S6) it was possible to carry out structural analysis, as their substituted residues are located in one of the domains for which 3D arrangement can be modeled (RAD21-SMC3 domain, RAD21-STAG domain and RAD21-SMC1A domain, Fig. 2; Figs. S2-3). Interactions between RAD21 and its binding partners are shown in Fig. S1.

#### Modeled missense variants within the RAD21-SMC3 domain (residues 18–87 harboring Arg65Gln), and RAD21-STAG domain (residues 321–392 harboring Ser345Pro, Pro355Leu and Pro376Arg)

Substitution of Arg65 with Gln (Arg65Gln) is a semi-conservative variation that did not promote detectable structural or dynamic changes in the complex. The Ser345Pro variant impairs RAD21 and STAG1/2 interactions due to promotion of a de novo curved small alpha-helix segment that binds to the pre-existing alpha helix, which separates from the surface of STAG2. No structural or dynamic effects of Pro355Leu or Pro367Arg on RAD21 itself could be observed. Nevertheless, Pro376Arg does promote the formation of a new salt bridge between RAD21 and STAG2, which is predicted to cause over-stabilization of the interaction between the two proteins.

#### Modeled missense variants within the RAD21-SMC1A domain (residues 543–628 harboring Gly575Ala, Cys585Arg, Arg586Gln, Gln592del, Phe600del, Leu603Pro, Ser618Gly, and Ala622Thr)

Four of the eight variants in this domain (Cys585Arg; Arg586Gln; Gln592del; Leu603Pro) are predicted to cause a structural effect. Arg586Gln destabilizes the RAD21-SMC1A domain by loss of a salt bridge between Arg586 and Glu577, and the altered position of Glu577 adds an additional negative charge to the RAD21 surface of RAD21-SMC1A. Cys585Arg has a similar effect, interacting with Glu583 and causing Arg586 to lose its contact with Glu577. The MD simulation shows that both Gln592del and Leu603Pro, but not Phe600del, affect the positioning of SMC1A-Asn35 at the ATPase site 1 by changing the position of Lys605.

### Phenotype

#### Physical features

**Fig. 3:**
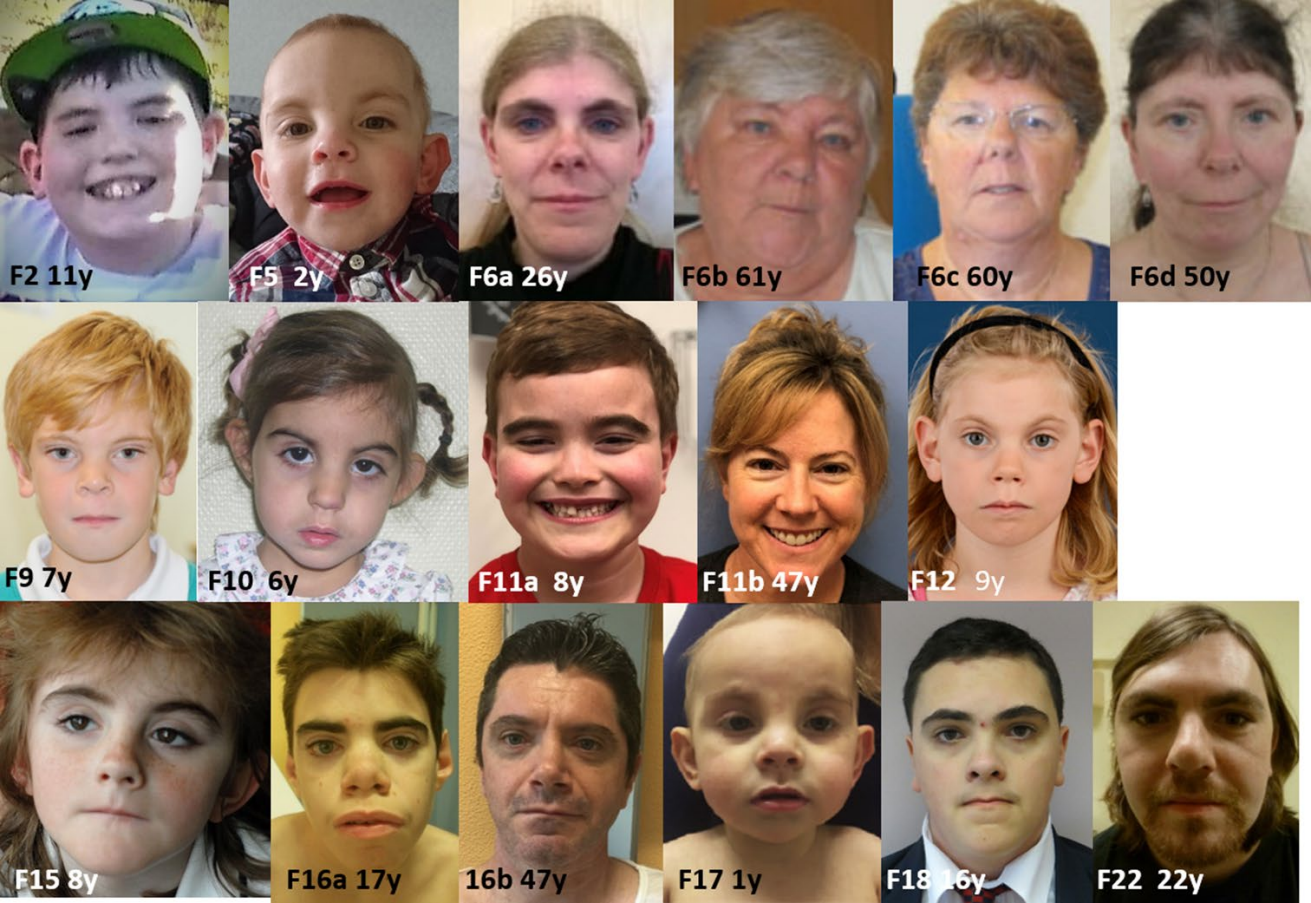
Clinical phenotype in *RAD21* patients. Anterior–posterior facial views. *F* family identification number, *y* age in years. Family numbers correspond to family numbers in the tables. Ages are indicated below each picture. Intrafamilial variability is illustrated by the comparison of facial morphology between the members of family F6 and of family F16. Interfamilial variability is illustrated by the comparison of facial morphology between patients F15 and F16a/b who harbor the p.(Arg586*) variant. Pictures of members of F6 and of F17 were republished with permission (Gudmundsson et al. 2018 and Boyle et al. 2017)

Individual CdLS scores and major and minor anomalies in cohort A are provided in Table S2-3. Clinical features of cohort A are compared to those of *NIPBL* and *SMC1A* cohorts in Table 2 and illustrated in Fig. 3 and Fig. S4. Clinical information for cohort B is available in supplemental materials S5 and will not be discussed further in the text, as clinical data are limited. We mention data in the text only if not represented in the tables.

**Table 2:** Comparison of clinical characteristics of present series of individuals with *RAD21* variants with sufficient clinical data (cohort A) with those in individuals with *SMC1A* and *NIPBL* variants [adapted from (Huisman et al. 2017)]

Clinical characteristics^a^	HPO ID	*RAD21* (*n* = 29)		*SMC1A* (*n* = 51)		*NIPBL* (*n* = 67)	
		*N* pos/*N* total	Percentage	*N* pos/*N* total	Percentage	*N* pos/*N* total	Percentage
Sex (male/female)		15/14	52/48	14/37	27/73	34/33	51/49
Familial mutation		5/12	42	4/47	9	n/a	n/a
Length at birth < − 2SD	HP:0003561	2/18	22	9/31	28	32/43	74
Weight at birth < − 2SD	HP:0001511	4/22	18	11/41	27	29/43	67
Prenatal head circumference < − 2SD	HP:0000252	7/16	44	8/24	33	39/43	91
Postnatal height < − 2SD	HP:0008897	10/27	37	24/38	63	37/43	86
Postnatal weight < − 2SD	HP:0004325	3/26	12	14/37	38	39/43	91
Postnatal head circumference < − 2SD	HP:0000252	16/28	57	23/36	64	54/62	87
Brachycephaly	HP:0000248	8/19	42	17/42	40	44/67	66
Low anterior/posterior hairline	HP:0000294/HP:0002162	14/23	61	30/43	70	57/67	85
Arched eyebrows	HP:0002553	18/27	67	32/44	73	54/67	81
Synophrys	HP:0000664	19/28	68	37/46	80	61/67	91
Thick eyebrows	HP:0000574	20/24	83	37/46	80	61/67	91
Long eyelashes	HP:0000527	21/26	81	38/45	84	65/67	97
Concave nasal ridge	HP:0011120	24/29	83	20/43	47	57/67	85
Upturned nasal tip	HP:0000463	19/27	70	26/46	57	58/67	87
Short nose	HP:0003196	23/26	88	26/46	57	58/67	87
Long and/or smooth philtrum	HP:0000343/HP:0000319	26/29	90	27/43	63	54/67	81
Thin upper lip vermillion	HP:0000219	23/29	79	33/44	75	22/24	92
Thin lips, downturned corners mouth	HP:0002714	16/27	59	33/46	72	23/24	96
Highly arched palate	HP:0000218	8/22	36	11/37	30	35/67	52
Cleft palate or submucous cleft palate	HP:0000175/HP:0410031	6/25	24	10/45	22	20/67	30
Widely spaced or absent teeth	HP:0000687/HP:0006349	2/20	10	13/44	30	18/23	78
Micrognathia	HP:0000347	8/23	35	18/45	40	50/67	75
Low-set and/or malformed ears	HP:0000369/HP:0000377	14/26	54	18/45	40	45/67	67
Major limb malformation	HP:0001180/HP:0009776	0/29	0	0/49	0	17/67	25
Small hands	HP:0200055	5/27	19	32/45	71	53/63	84
Proximally placed thumb	HP:0009623	6/18	33	18/44	41	11/20	55
Clinodactyly 5th finger	HP:0004209	13/24	54	21/45	47	42/63	67
Short 5th finger	HP:0009237	23/28	82	21/45	47	42/63	67
Syndactyly hands	HP:0006101	1/19	5	1/37	3	4/63	6
Abnormal palmar crease	HP:0010490	9/21	43	5/40	13	21/29	72
Dislocated elbow/abnormal extension	HP:0005021/HP:0001377	11/24	46	2/40	5	20/34	59
Small feet	HP:0001773	3/27	11	29/44	66	65/67	97
Syndactyly 2nd–3rd toes	HP:0004691	4/24	17	13/46	28	21/66	32
Scoliosis	HP:0002650	2/20	10	4/40	10	1/42	2
Hip dislocation or dysplasia	HP:0002827/HP:0001385	2/19	11	2/40	5		
Ptosis	HP:0000508	11/26	42	4/40	10	8/42	19
Visual impairment	HP:0000505	0/24	0	20/38	53	29/66	44
Myopia ≥ − 6.00 D	HP:0011003	≤ 2/24^b^	≤8	11/40	28	6/40	15
Hearing loss	HP:0000365	8/24	33	16/39	41	43/66	65
Seizures	HP:0001250	2/22	9	20/44	45	10/66	15
Cutis marmorata	HP:0000965	3/23	13	19/44	43	27/43	63
Hirsutism	HP:0001007	10/26	38	37/47	79	37/43	86
CNS major and minor malformations (MRI brain)	HP:0012443	2/5	40	5/43	12		
Heart (major and minor)	HP:0001627	9/23	39	13/44	30	18/66	27
Major malformation of gut	HP:0012718	4/30	13	3/44	7	6/24	25
Diaphragmatic hernia	HP:0000776	1/30	3	1/40	3	6/24	25
Gastroesophageal reflux disease	HP:0002020	13/25	52	25/42	60	47/66	71
Genitourinary system major^c^	HP:0000119	1/20	5	4/42	10	0/67	0
Genitourinary system minor	HP:0000119	8/23	35	9/40	23	46/67	69

*HPO ID* human phenotype ontology identifier, *CNS* central nervous system

^a^Only features which could be compared across at least two cohorts are presented. Full clinical description with individual data are presented in supplementary Table S3

^b^2 of the 24 cases have myopia but unspecified severity

^c^Uni/bilateral renal anomalies

All patients in cohort A (age range 0–61 years, median 9 years, mean 18 years; 15 males) had CdLS scores of at least five, sufficient to warrant molecular genetic testing for CdLS. In about 60% of index cases (13/21 index cases in which this was specified) CdLS was suspected prior to testing. There was no gender difference in CdLS scores. No *RAD21* variant would have been missed using the CdLS consensus criteria for molecular studies (Kline et al. 2018). Clinical scores of patients with CdLS suspected prior to testing (median 11.5; range 8–13) were higher than those not suspected to have CdLS (median 9.5; range 5–13).

#### Cognition, development and behavior

Cognitive functioning, developmental milestones and behavioral functioning in the *RAD21* group are attenuated compared to the *NIPBL* and *SMC1A* groups (Tables 3 and S4). The majority of *RAD21* patients (16/29, 55%) have normal or mildly impaired cognitive functioning (*SMC1A* group 32%; *NIPBL* group 7%) (Huisman et al., 2017; Mulder et al., 2019). In all three groups, there is a trend towards more language-based problems than motor-based problems in development. Still, all *RAD21* patients aged 3 years and above were able to use some words. There was no correlation between the severity of cognitive impairment in *RAD21* patients and presence of microcephaly (prenatal, postnatal, or both; data not shown).

**Table 3:** Cognitive and behavioral characteristics of individuals with *RAD21* variants with sufficient clinical data (cohort A) with those in individuals with *SMC1A* and *NIPBL* variants [adapted from (Huisman et al. 2017; Moss et al. 2017; Mulder et al. 2019)]

	*RAD21* (*n* = 29)		*SMC1A* (*n* = 51)		*NIPBL* (*n* = 67)	
	*N* pos/*N* total	%	*N* pos/*N* total	%	*N* pos/*N* total	%
Cognitive functioning^a^						
Normal cognition	3/29^b^	10	3/28	11	0/58	0
Mild disability (HP:0001256)	13/29	45	6/28	21	4/58	7
Moderate disability (HP:0002342)	4/29^c^	14	9/28	32	16/58	28
Severe disability (HP:0010864)	0/29	0	6/28	21	27/58	47
Profound disability (HP:0002187)	0/29	0	4/28	14	11/58	19
Disability present, severity unspecified (HP:0001249)	2/29	7				
Developmental problems, too young to determine reliably cognitive functioning (HP:0012759)	7/29	24				
Developmental milestones^d^						
Sitting without support		100^e^		75^e^		54^e^
Attained on target (age < 12 months)	10/10		n/a		n/a	
Attained before age 3 years	10/10		18/24		28/52	
Attained later			3/24		23/52	
Not attained yet (in patients aged ≥ 5 years)			3/24		1/52	
First words		100^e^		35^e^		8^e^
Attained on target (age < 15 months)	6/15		n/a		n/a	
Attained before age 3 years	15/15		7/20		4/53	
Attained later			4/20		16/53	
Not attained yet patients aged ≥ 5 years)			9/20		33/53	
Walking without support		100^e^		57^e^		≥29^e,g^
Attained on target (age < 18 months)	12/16		n/a		1/52	
Attained before age 3 years	16/16		17/30		2/52	
Attained later			9/30		12/52	
Not attained yet (in patients aged ≥ 5 years)			4/30		19/52	
Delay on one or more milestone	12/16	75	18/20	90	51/52	98
Behavior^f^						
Attention deficit disorder ± hyperactivity	8/23	35				
Obsessive–compulsive behavior	6/19	32	10/26^h^	38		
Anxiety	10/19	53				
Constant roaming	3/15	20				
Aggression	1/16	6			12/15^h^	80
Self-injurious behavior	1/18	6	11/31	35	47/61	77
Extreme shyness or withdrawal	0/17	0				
Autistic-like features	7/20	35	18/31^h^	56	9/13^h^	69
One or more behavioral domains affected	14/25	56				

*HP* human phenotype ontology identifier

^a^RAD21, 8 formal test results, others physician reported data. Equivalent HP is shown between brackets

^b^Includes 2 adults with learning disabilities but reported normal cognitive functioning

^c^Including 2 moderate/severe

^d^Only scored if child was older than target age

^e^Percentage of individuals that attain the milestone before age 3 years

^f^RAD21: 5 formal test results, others physician-reported data

^g^Including 18 patients that attained the milestone late, but age unknown

^h^Based on formal testing

14/25 *RAD21* patients (56%) with sufficiently available information on behavior had problems, mainly features of anxiety, ADHD, ASD, and obsessive–compulsive behavior. ASD related problems, aggression and SIB were less prevalent compared to the *SMCIA* and *NIPBL* groups.

### Genotype–phenotype comparisons in cohort A

#### Microdeletions versus intragenic variants

There was a trend towards higher CdLS scores and more frequently impaired growth parameters in patients with microdeletions compared to those with intragenic variants, but no differences were apparent in frequency of major malformations or cognitive and behavioral problems. We refrained from statistical analyses as small numbers would make results too unreliable and less useful. Exostoses, related to *EXT1* haploinsufficiency, likely caused the upper limb anomalies.

#### Truncating versus non-truncating sequence variants

There was no difference in CdLS scores or growth parameters between individuals with truncating and those with non-truncating sequence variants (median 10; range 9–13 and median 9.5; range 5–12, respectively).

#### Malformations and genotype

For 12/15 patients with intragenic variants and major malformations or health problems, the variant was located in a protein-binding domain (F2, F3a, F8, F9, F11a, F11b, F12, F14a, F14b, F16a, F17, F18). As numbers are small it remains uncertain whether this is truly an association. The types of major malformations did not differ.

#### Intrafamilial variation

The intrafamilial variation can be considerable (Tables S1, S3-4; Fig. 3), especially in cognition and behavior. Through obvious ascertainment bias cognition is more frequently impaired in index cases. Several families include patients with ID and patients with apparently normal cognitive functioning. The intrafamilial variation cannot be explained by mosaicism in most families.

## Discussion

We report on *RAD21* variants in 49 individuals, some with sufficient clinical data (cohort A), others with limited clinical data (cohort B). *RAD21* variants are frequently familial, often unique, and without obvious hotspots for variants or microdeletions breakpoints, although missense variants tend to cluster around protein binding domains.

### *RAD21* missense variants and their predicted effect on protein function

The structural and functional analysis indicated that at least six out of twelve modeled *RAD21* missense variants are likely pathogenic (Ser345Pro, Pro367Arg, Cys585Arg, Arg586Gln (reported as a VUS), Gln592del and Leu603Pro). If phenotype data and literature/database information are taken into account, three more *RAD21* modeled missense variants are likely pathogenic (Arg65Gln (reported as VUS), Phe600del, Ala622Thr).

The Arg65 is located within the RAS21-SMC3 domain in the close proximity of Tyr67, and altering the kinase/phosphatase recognition motif Arg-X-Tyr around Tyr67 may affect the phosphorylation-based regulation of RAD21 (Amanchy et al. 2011; Hoque and Ishikawa 2001; Hornbeck et al. 2015; Li et al. 2009). In addition, a contact between the PDS5 protein and the RAD21-SMC3/SMC3-head complex is involved in the topological entrapment of DNA by cohesin (Guacci et al. 2019). As Arg65 is located towards the solvent, Arg65Gln may impact the RAD21-PDS5 recognition and, thus, disturb their interaction.

The interaction between RAD21 and STAG1/2 is crucial for the proper functioning of the cohesin complex (Guacci et al. 2019), and both impairing (Ser345Pro) or over-stabilizing (Pro367Arg) variants within the RAD21-STAG domain are predicted to cause dysfunction of the complex, presumably through affecting the continuous cycle of formation and disengagement of the cohesin ring (Marcos-Alcalde et al. 2017).

The structural model of the RAD21-SMC1A domain rationalizes the key function of RAD21 in the ATPase reaction at the SMC1A/SMC3 head, which is pivotal to the opening of the cohesin ring, and thus the cyclic process (Marcos-Alcalde et al. 2017). The Cys585Arg and Arg586Gln variants destabilize the RAD21-SMC1A domain; and Gln592del and Leu603Pro (but not Phe600del) disturb the cyclic process through the dislocation of Lys605. Although the Phe600del variant does not seem to affect RAD21 structure, it leads to a classical CdLS phenotype without variants in additional known CdLS genes (using a targeted gene panel). Thus, it does seem likely pathogenic. Unfortunately, the crystal structure of RAD21 is not available for other domains or interacting partners such as WAPL and PDS5, but earlier molecular studies provide additional information for other missense variants.

The importance of the regulation of the interaction between RAD21-SMC1A and SMC1A/SMC3 head is demonstrated by the several residues involved in phosphorylation and ubiquitination in the RAD21-SMC1A domain (Hegemann et al. 2011; Hoque and Ishikawa 2001; Hornbeck et al. 2015). Ala622 is positioned next to Thr623, a substrate for protein phosphorylation by PLK1 (Hornbeck et al. 2015; Tsai et al. 2015). A pathogenic effect of variant Ala622Thr is supported by studies showing decreased bowel transit and loss of enteric neurons in zebrafish with Ala622Thr knockdown through morpholinos and by patients with biallelic Ala622Thr variants and Mungan syndrome with CIPO (chronic intestinal pseudo-obstruction) (Bonora et al. 2015). The heterozygous members of this family had some clinical features of the CdLS spectrum, but as it was not possible to retrieve further clinical data, it remains uncertain whether they have a full CdLS phenotype, and whether this variant can lead to a phenotype in heterozygous form.

For two additional variants that could not be modeled, the literature supports that they are likely pathogenic. Phe6 is found close to Ser9, a phosphorylation site described in the human proteome (Gauci et al. 2009; Guacci et al. 2019). The Phe6Val variant (reported as aVUS) would modify the kinase/phosphatase recognition motif, thus affecting the protein behavior. Similarly, as residue Thr461 is flanked by Ser residues (Ser459 and Ser466), both implicated in phosphorylation-regulated dissociation of cohesin from chromosome arms (Hauf et al. 2005; Hornbeck et al. 2015), it may modify the kinase/phosphatase recognition motif.

### Clinical phenotype

#### Physical phenotype

*RAD21* variants can lead to a CdLS phenotype (*RAD21*-CdLS). The (limited) available information of individuals from cohort B suggests that biallelic *RAD21* variants can also lead to Mungan syndrome and monoallelic *RAD21* variants to holoprosencephaly (like one case in cohort A) and possibly schizophrenia, although in the latter the association may be a spurious coincidence. In Table S6 we describe several additional cases with phenotypes including sclerocornea and schizophrenia, in which pathogenicity of the *RAD21* variant is debatable. Due to incomplete information it remains uncertain whether these individuals are also showing CdLS characteristics. Indeed, when we succeeded in obtaining further clinical information, several individuals turned out to show CdLS characteristics not mentioned in the publication (for instance in the family with Mungan syndrome). Additionally, one may speculate that phenotypes are also attributable (possibly in addition to the *RAD21* variant) to variants in other genes.

#### Comparison to phenotypes of NIPBL and SMC1A variants

In patients with sufficient clinical data available (cohort A) most features associated with CdLS are present. However, the prevalence of features is lower compared to those in the *SMC1A* and *NIPBL* cohort, and the degree of severity is typically less. Severe visual impairment and diaphragmatic hernias are rare in *RAD21* patients, and feeding difficulties are uncommon. *RAD21* patients less frequently have increased body hair (hirsutism, bushy eyebrows, low scalp hair lines), major limb malformations are not reported, and hands and feet are generally of normal size. Still, minor anomalies of hands and feet are common, such as fetal pads, abnormal flexion crease patterns, and camptodactyly. Patients with *RAD21* variants have generally less impaired growth at birth, and short stature and microcephaly develop postnatally. Prenatal microcephaly has been demonstrated to be a predictor of more severe cognitive impairment in CdLS in the pre-molecular era (Hawley et al. 1985) but this does not hold for *RAD21* patients. Frequency and severity of congenital heart defects are similar to those in the *NIPBL* and *SMC1A* cohorts. Gastro-esophageal reflux is similar in frequency but in *RAD21* it is typically mild and restricted to early childhood. No *RAD21* patients exhibit a Rett-like phenotype as can occur in a subgroup of patients with *SMC1A* variants (Huisman et al. 2017). The CdLS score remains a reliable tool, and the present study does not call for an adjustment of the diagnostic advice from the CdLS guidelines (Kline et al. 2018).

Unusual anomalies in the *RAD21* cases are vertebral anomalies (clefts and hemivertebrae). There is a single individual with a *NIPBL* variant and Klippel–Feil anomaly (personal observation RCH), and upper cervical spine malformations have been reported in other patients with *NIPBL* variants as well (Bettini et al. 2014). Malformations of structures derived from the embryonic foregut are relatively frequent in *RAD21* patients and have only rarely been described in CdLS (Hamilton et al. 2014; Kang et al. 2018; Mende et al. 2012). Holoprosencephaly spectrum anomalies have been linked to several cohesin genes (Kruszka et al. 2019), including *RAD21*, although in one individual this remains uncertain (Table S6). The prevalence of holoprosencephaly spectrum in *RAD21*-CdLS must remain uncertain as brain MRIs are typically not indicated in individuals with CdLS due to the burden of the procedure and lack of consequences of findings for care (Kline et al. 2018).

#### Development, cognition and behavior

Most data on cognition and behavior in the present cohort are based on subjective information provided by physicians and not on formal testing. Therefore, reliability remains uncertain. Still, all data point to a lower prevalence and decreased severity of ID in *RAD21* patients compared to *NIPBL* and *SMC1A* groups: developmental milestones are more frequently attained, the cognitive level is estimated higher, and aggression and autism are less frequent. SIB, a hallmark of CdLS in general (Kline et al. 2018), is infrequent in *RAD21* individuals.

Even if an IQ is normal, subtle difficulties in neuropsychological domains known to be affected in CdLS (Kline et al. 2018) may influence cognitive performance. Periodic formal screening for neuropsychological and behavioral problems is still warranted in all individuals with *RAD21* variants, to allow for early recognition of problems and access to relevant support systems. In addition, formal (in-person) assessments can prevent misdiagnoses, such as autism, by putting behavioral characteristics into the perspective of the developmental level of patients (Mulder et al. 2019).

#### Natural history

The natural history data from the present study indicate that pregnancies and birth tend to progress normally, prenatal growth retardation being present in a small minority. About half of the patients have congenital anomalies (cleft palate; cardiac anomalies). Major limb defects have not been found; diaphragmatic hernia, anal atresia or choanal atresia occur occasionally. Patients have typically mild facial dysmorphisms, no small hands or feet, and increased body hair is less apparent compared to *SMC1A* and *NIPBL* patients. The clinical diagnosis of CdLS may, therefore, be difficult.

Neonatal feeding is usually not problematic. Reflux is common but not severe. Typical development is somewhat slow, mainly in speech development, and physical therapy or speech therapy may be indicated. As they grow up, children only occasionally develop new medical problems. Half of the children show a progressive but still mild growth delay in head circumference and height. Vision is mostly normal; hearing loss is found in a third of individuals and may require hearing devices. Most of the patients are able to attend regular education or education for children with mild cognitive disabilities. Most have some behavioral problems (mainly anxiety, ADHD or ASD) of limited severity, and aggression and SIB are uncommon. Not uncommonly, *RAD21* patients are able to start a family, and some are only diagnosed when more severely affected offspring is recognized. This indicates that careful family analysis is paramount in each family in which someone is diagnosed with a *RAD21* variant.

### Genotype–phenotype associations

The relatively mild phenotype of patients with *RAD21* variants seems to indicate that RAD21 is not highly intolerant to loss-of-function, in contrast to other CdLS-associated genes (*NIPBL, SMC1A, PDS5, WAPL, STAG2*) (Gause et al. 2010). Supporting this, Deardorff et al. found haploinsufficiency for *RAD21* led to approximately halved RAD21 RNA in a cell line from a patient with classical CdLS, while haploinsufficiency for *NIPBL* is often associated with a compensatory upregulation of RNA levels, presumably from the intact allele (Borck et al. 2006; Deardorff et al. 2012; Newkirk et al. 2017). One may speculate that the effect of haploinsufficiency of *RAD21* on the function of the cohesin ring can be compensated more effectively compared to the other cohesin genes. However, patients with haploinsufficiency for *RAD21* due to microdeletions or truncating variants do not differ markedly from those with missense variants, and nonsense-mediated decay is not apparent although in the present series of patients this was not formally tested. It remains uncertain whether duplication of the whole gene can lead to a CdLS phenotype, as demonstrated for duplications in *STAG2* and *SMC1A* (Baquero-Montoya et al. 2014; Mullegama et al. 2019), as all duplications we retrieved, were either including several other genes or pathogenicity could not be confirmed. No fully intragenic duplication is known to us. Small duplications have also been detected in apparently healthy controls (unpublished observations J. Howe).

In general, protein studies combined with a detailed phenotype allow often, but not always, to probe for RAD21 dysfunction in patients with variants of doubtful meaning. The phenotype in individuals with *RAD21* variants is not only determined by the variant itself but potentially also by other factors: (1) variable expression of cohesin subunits and/or binding partners in different tissues; (2) variable formation of isoforms in different tissues; (3) modifying genes, especially of the cohesin complex (Yuan et al. 2019); (4) epigenetic factors such as DNA methylation and gene silencing (Aref-Eshghi et al. 2019), exogenous influences including support and education, and other factors such as host-microbiome interactions. Exact phenotypic consequences, if any, of each of the above are unknown. Specifically epigenetic influences may be important. Genome-wide methylation patterns (epi-signatures) have been shown to be altered in CdLS (Aref-Eshghi et al. 2020). Likely, complex interactions between several of the above factors play a role.

In counseling of families with *RAD21* variants, the relatively high frequency of familial occurrence and marked intrafamilial and interfamilial variability should be mentioned. Parental testing is warranted, even if signs or symptoms are apparently absent in parents, and standard testing of parents may further broaden the phenotype of *RAD21* variants. We suggest a cautious use of data on variants in molecular databases, as due to the extremely variable and sometimes very mild phenotype wrong conclusions may be drawn in classifying the variants. In case of a CdLS phenotype and detection of a VUS in *RAD2*1 in which pathogenicity cannot be determined using clinical and molecular data of the parents, we recommend testing for variants in other CdLS associated genes and eventually carry out ‘open’ exome/genome sequencing to rule out variants in other genes.

### Limitations

Although we used a broad search strategy and the present *RAD21* cohort is the largest reported thus far, numbers are still small, and these preclude further statistical analyses. We did not consider variants from ClinVar or Decipher that were reportedly (likely) benign, but we expect that these may contain some pathogenic variants discarded based on an overlooked (mild) phenotype. Furthermore, many variants are reported with insufficient clinical data preventing such patients to be included in the present series. Especially morphological data are often missing, and we stress the importance of the use of the CdLS consensus data in evaluating individuals with variants in cohesin genes (Kline et al. 2018). Next generation sequencing-based technologies such as gene panels or ‘open’ exome/genome sequencing remains to be introduced in many countries, and we expect identification of many additional patients with pathogenic *RAD21* variants as clinical recognition may be difficult. Finally, we may have an acquisition bias due to the involvement of specialists in CdLS, causing an overrepresentation of individuals with a CdLS phenotype.

### Future

The present results demonstrate that more information on larger groups of individuals with *RAD21* variants is needed to determine the complete phenotypic spectrum. CdLS characteristics such as sleep disturbances and autonomic dysfunctions in individuals with *RAD21* variants are still largely unknown. A specific issue that needs attention is the risk to develop cancer (incidentally reported to date in RAD21 patients) (Deardorff et al. 2012; Minor et al. 2014). We call also for more detailed study of cognitive, behavioral and psychiatric phenotypes, as these are of utmost importance in clinical care. Molecular and cellular mechanisms underlying cognitive problems are unclear, although cohesin-mediated 3D-organization of the genome is suggested to play a role in neuronal plasticity (Fujita and Yamashita 2018). Studying RAD21 and other cohesin components in this process could contribute to the search for targeted influencing of cognition and especially behavior in CdLS. Effects of *RAD21* variants on cellular functioning and relationships between genotype and phenotype may be elucidated further by studying epi-signatures. This may explain presently unexpected discrepancies between genotype and phenotype, and even allow for establishing pathogenicity in individuals with uncertain molecular findings.

## Methods

### Patients

Patients were gathered using a combination of literature and database search and network inquiries (see Supporting Information). A dedicated questionnaire was used to gather clinical, molecular, cognitive and behavioral data. If allowed by the family clinical pictures were gathered for the scoring of facial characteristics by the senior author (RCH). If no clinical pictures were available to us (*n* = 3 in cohort A) the clinician-reported description of facial characteristics was accepted. The CdLS clinical score (reflecting the similarity of clinical features to those in classical CdLS) was computed using cardinal features (2 points each) and suggestive features (1 point each) according to Kline et al. (2018).

Information on cognitive functioning and behavioral problems was derived from physician-reported data, if possible substantiated with results of formal testing. For the CdLS clinical score, minor criterion “ID or global DD” was scored positive if ID or global DD (global developmental delay; a combination of delay in at least 2 developmental domains) was present, at any age. Elsewhere in the manuscript, cognitive functioning has been classified into categories based on DSM-5.

To compare the *RAD21* phenotype to CdLS patients with variants in other genes, clinical data were obtained from existing *NIPBL* and *SMC1A* cohorts (Huisman et al. 2017; Mulder et al. 2019), to which we added further information if needed. For comparison of features of ASD and aggression in *NIPBL* patients, we derived information from the UK cohort (Moss et al. 2017). For the item ‘autistic like behavior’, we compared with scores from the Social Communication Questionnaire (number above cut-off for ASD); for the item ‘aggression’ with presence of verbal aggression, physical aggression or property destruction on the Challenging Behavior Questionnaire; and for ‘obsessive–compulsive behavior’ with the number of patients with one or more items of the compulsive behavior subscale of the Repetitive Behavior Questionnaire above clinical cutoff.

Based on the availability of clinical data, we composed two cohorts: cohort A with sufficient clinical data available on all cardinal CdLS features, and cohort B with incomplete clinical data. We provide an overview of excluded cases in Supporting Information Table S6.

### Molecular studies

Among the 29 patients (22 index) of cohort A, a clinical diagnosis of CdLS was suspected in 13 index cases, which allowed detection of *RAD21* variants using array comparative genomic hybridization [CGH (*n* = 1), Sanger sequencing (*n* = 6), ‘whole’ exome sequencing (WES, *n* = 2), or targeted exome sequencing searching for variants in genes that can cause intellectual disability (ID-WES, *n* = 4)]. Confirmation by Sanger sequencing of an exome result was only performed if the coverage of the exome was thought to be of insufficient quality. The other nine index cases were detected through Sanger sequencing of a series of candidate genes after excluding a clinical diagnosis (KBG syndrome, *n* = 1), or after WES (*n* = 2), ID-WES (*n* = 3), or array CGH or SNP array (*n* = 3). All molecular studies were performed for diagnostic reasons, following the various national regulations, and for none of the patients studies were performed because of the current research. For describing the variants coding DNA reference sequence NM_006265.2(*RAD21*_v001) is used.

### Structure modeling of RAD21 variants

We checked the predicted effect of all missense variants retrieved with the splice prediction tool of the Alamut software (https://www.interactive-biosoftware.com/alamut-visual/). We proceeded with all variants which could be modelled regardless of their reported classification to retrieve further evidence for their effect (or lack of it) on protein function.

A set of three wild-type and twelve variant protein models was generated through standard homology modeling procedures using the SWISS-MODEL server (http://swissmodel.expasy.org; see Supporting Information). These were used to study the structural effects of the missense variants located in the protein domains in contact with SMC3 (RAD21 N-terminus; RAD21-SMC3), STAG1/2 (RAD21-STAG) or SMC1A (RAD21 C-terminus, RAD21-SMC1A).

### Molecular dynamics simulations

To analyze the putative effect of variants on the RAD21 structure, the behavior of the 12 variant proteins were compared to that of wild type models by free molecular dynamics (MD) simulation for 60–100 ns (ns) (see Supporting Information). Movements during the trajectories were continuously measured by root-mean square deviation (RMSD) of atomic positions. Large variations of RMSD values indicate notable distortions of protein structure due to the abnormal amino acid variant. RAD21 domains were modeled in complex with the accompanying proteins, to facilitate functional evaluation of variants along the MD trajectories.

### Ethics

All clinical investigation has been conducted according to Declaration of Helsinki principles. Written informed consent was received from participants prior to inclusion in the study. Patients or their legal representatives have provided written consent for using images. The Medical Ethics Committee of the Amsterdam UMC approved the study (NL39553.018.12).

## Electronic supplementary material

Below is the link to the electronic supplementary material.

Supplementary material 1 (PDF 1941 kb)

## References

[CR1] Amanchy R, Kandasamy K, Mathivanan S, Periaswamy B, Reddy R, Yoon WH, Joore J, Beer MA, Cope L, Pandey A (2011). Identification of novel phosphorylation motifs through an integrative computational and experimental analysis of the human phosphoproteome. J Proteomics Bioinform.

[CR2] Ansari M, Poke G, Ferry Q, Williamson K, Aldridge R, Meynert AM, Bengani H, Chan CY, Kayserili H, Avci S, Hennekam RC, Lampe AK, Redeker E, Homfray T, Ross A, Falkenberg Smeland M, Mansour S, Parker MJ, Cook JA, Splitt M, Fisher RB, Fryer A, Magee AC, Wilkie A, Barnicoat A, Brady AF, Cooper NS, Mercer C, Deshpande C, Bennett CP, Pilz DT, Ruddy D, Cilliers D, Johnson DS, Josifova D, Rosser E, Thompson EM, Wakeling E, Kinning E, Stewart F, Flinter F, Girisha KM, Cox H, Firth HV, Kingston H, Wee JS, Hurst JA, Clayton-Smith J, Tolmie J, Vogt J, Tatton-Brown K, Chandler K, Prescott K, Wilson L, Behnam M, McEntagart M, Davidson R, Lynch SA, Sisodiya S, Mehta SG, McKee SA, Mohammed S, Holden S, Park SM, Holder SE, Harrison V, McConnell V, Lam WK, Green AJ, Donnai D, Bitner-Glindzicz M, Donnelly DE, Nellaker C, Taylor MS, FitzPatrick DR (2014). Genetic heterogeneity in Cornelia de Lange syndrome (CdLS) and CdLS-like phenotypes with observed and predicted levels of mosaicism. J Med Genet.

[CR3] Aref-Eshghi E, Bend EG, Colaiacovo S, Caudle M, Chakrabarti R, Napier M, Brick L, Brady L, Carere DA, Levy MA, Kerkhof J, Stuart A, Saleh M, Beaudet AL, Li C, Kozenko M, Karp N, Prasad C, Siu VM, Tarnopolsky MA, Ainsworth PJ, Lin H, Rodenhiser DI, Krantz ID, Deardorff MA, Schwartz CE, Sadikovic B (2019). Diagnostic utility of genome-wide DNA methylation testing in genetically unsolved individuals with suspected hereditary conditions. Am J Hum Genet.

[CR4] Aref-Eshghi E, Kerkhof J, Pedro VP, Barat-Houari M, Ruiz- Pallares N, Andrau JC, Lacombe D, Van-Gils J, Fergelot P, Dubourg C, Sadikovic B (2020) Evaluation of DNA methylation EpiSigns for diagnosis and phenotype correlations in 42 Mendelian neurodevelopmental disorders. Am J Hum Genet **(in press)** 10.1016/j.ajhg.2020.01.019 PMC7058829 32109418

[CR5] Baquero-Montoya C, Gil-Rodriguez MC, Teresa-Rodrigo ME, Hernandez-Marcos M, Bueno-Lozano G, Bueno-Martinez I, Remeseiro S, Fernandez-Hernandez R, Bassecourt-Serra M, Rodriguez de Alba M, Queralt E, Losada A, Puisac B, Ramos FJ, Pie J (2014). Could a patient with SMC1A duplication be classified as a human cohesinopathy?. Clin Genet.

[CR6] Bettini LR, Locatelli L, Mariani M, Cianci P, Giussani C, Canonico F, Cereda A, Russo S, Gervasini C, Biondi A, Selicorni A (2014). Cervical spine malformation in Cornelia de Lange syndrome: a report of three patients. Am J Med Genet A.

[CR7] Bonora E, Bianco F, Cordeddu L, Bamshad M, Francescatto L, Dowless D, Stanghellini V, Cogliandro RF, Lindberg G, Mungan Z, Cefle K, Ozcelik T, Palanduz S, Ozturk S, Gedikbasi A, Gori A, Pippucci T, Graziano C, Volta U, Caio G, Barbara G, D’Amato M, Seri M, Katsanis N, Romeo G, De Giorgio R (2015). Mutations in RAD21 disrupt regulation of APOB in patients with chronic intestinal pseudo-obstruction. Gastroenterology.

[CR8] Borck G, Zarhrate M, Cluzeau C, Bal E, Bonnefont JP, Munnich A, Cormier-Daire V, Colleaux L (2006). Father-to-daughter transmission of Cornelia de Lange syndrome caused by a mutation in the 5’ untranslated region of the NIPBL Gene. Hum Mutat.

[CR9] Boyle MI, Jespersgaard C, Nazaryan L, Bisgaard AM, Tumer Z (2017). A novel RAD21 variant associated with intrafamilial phenotypic variation in Cornelia de Lange syndrome—review of the literature. Clin Genet.

[CR10] Deardorff MA, Wilde JJ, Albrecht M, Dickinson E, Tennstedt S, Braunholz D, Monnich M, Yan Y, Xu W, Gil-Rodriguez MC, Clark D, Hakonarson H, Halbach S, Michelis LD, Rampuria A, Rossier E, Spranger S, Van Maldergem L, Lynch SA, Gillessen-Kaesbach G, Ludecke HJ, Ramsay RG, McKay MJ, Krantz ID, Xu H, Horsfield JA, Kaiser FJ (2012). RAD21 mutations cause a human cohesinopathy. Am J Hum Genet.

[CR11] Dorval S, Masciadri M, Mathot M, Russo S, Revencu N, Larizza L (2019). A novel RAD21 mutation in a boy with mild Cornelia de Lange presentation: Further delineation of the phenotype. Eur J Med Genet.

[CR12] Fisher JB, Peterson J, Reimer M, Stelloh C, Pulakanti K, Gerbec ZJ, Abel AM, Strouse JM, Strouse C, McNulty M, Malarkannan S, Crispino JD, Milanovich S, Rao S (2017). The cohesin subunit Rad21 is a negative regulator of hematopoietic self-renewal through epigenetic repression of Hoxa7 and Hoxa9. Leukemia.

[CR13] Fujita Y, Yamashita T (2018). Spatial organization of genome architecture in neuronal development and disease. Neurochem Int.

[CR14] Gauci S, Helbig AO, Slijper M, Krijgsveld J, Heck AJ, Mohammed S (2009). Lys-N and trypsin cover complementary parts of the phosphoproteome in a refined SCX-based approach. Anal Chem.

[CR15] Gause M, Misulovin Z, Bilyeu A, Dorsett D (2010). Dosage-sensitive regulation of cohesin chromosome binding and dynamics by Nipped-B, Pds5, and Wapl. Mol Cell Biol.

[CR16] Guacci V, Chatterjee F, Robison B, Koshland DE (2019). Communication between distinct subunit interfaces of the cohesin complex promotes its topological entrapment of DNA. Elife.

[CR17] Gudmundsson S, Anneren G, Marcos-Alcalde I, Wilbe M, Melin M, Gomez-Puertas P, Bondeson ML (2018). A novel RAD21 p.(Gln592del) variant expands the clinical description of Cornelia de Lange syndrome type 4—review of the literature. Eur J Med Genet.

[CR18] Hamilton J, Clement WA, Kubba H (2014). Otolaryngological presentations of Cornelia de Lange syndrome. Int J Pediatr Otorhinolaryngol.

[CR19] Hauf S, Roitinger E, Koch B, Dittrich CM, Mechtler K, Peters JM (2005). Dissociation of cohesin from chromosome arms and loss of arm cohesion during early mitosis depends on phosphorylation of SA2. PLoS Biol.

[CR20] Hawley PP, Jackson LG, Kurnit DM (1985). Sixty-four patients with Brachmann-de Lange syndrome: a survey. Am J Med Genet.

[CR21] Hegemann B, Hutchins JR, Hudecz O, Novatchkova M, Rameseder J, Sykora MM, Liu S, Mazanek M, Lenart P, Heriche JK, Poser I, Kraut N, Hyman AA, Yaffe MB, Mechtler K, Peters JM (2011). Systematic phosphorylation analysis of human mitotic protein complexes. Sci Signal.

[CR22] Hoque MT, Ishikawa F (2001). Human chromatid cohesin component hRad21 is phosphorylated in M phase and associated with metaphase centromeres. J Biol Chem.

[CR23] Hornbeck PV, Zhang B, Murray B, Kornhauser JM, Latham V, Skrzypek E (2015). PhosphoSitePlus, 2014: mutations, PTMs and recalibrations. Nucleic Acids Res.

[CR24] Huisman S, Mulder PA, Redeker E, Bader I, Bisgaard AM, Brooks A, Cereda A, Cinca C, Clark D, Cormier-Daire V, Deardorff MA, Diderich K, Elting M, van Essen A, FitzPatrick D, Gervasini C, Gillessen-Kaesbach G, Girisha KM, Hilhorst-Hofstee Y, Hopman S, Horn D, Isrie M, Jansen S, Jespersgaard C, Kaiser FJ, Kaur M, Kleefstra T, Krantz ID, Lakeman P, Landlust A, Lessel D, Michot C, Moss J, Noon SE, Oliver C, Parenti I, Pie J, Ramos FJ, Rieubland C, Russo S, Selicorni A, Tumer Z, Vorstenbosch R, Wenger TL, van Balkom I, Piening S, Wierzba J, Hennekam RC (2017). Phenotypes and genotypes in individuals with SMC1A variants. Am J Med Genet A.

[CR25] Kamada K, Barilla D (2018). Combing chromosomal DNA mediated by the SMC complex: structure and mechanisms. Bioessays.

[CR26] Kang MJ, Ahn SM, Hwang IT (2018). A novel frameshift mutation (c.5387_5388insTT) in NIPBL in Cornelia de Lange syndrome with severe phenotype. Ann Clin Lab Sci.

[CR27] Kline AD, Moss JF, Selicorni A, Bisgaard AM, Deardorff MA, Gillett PM, Ishman SL, Kerr LM, Levin AV, Mulder PA, Ramos FJ, Wierzba J, Ajmone PF, Axtell D, Blagowidow N, Cereda A, Costantino A, Cormier-Daire V, FitzPatrick D, Grados M, Groves L, Guthrie W, Huisman S, Kaiser FJ, Koekkoek G, Levis M, Mariani M, McCleery JP, Menke LA, Metrena A, O’Connor J, Oliver C, Pie J, Piening S, Potter CJ, Quaglio AL, Redeker E, Richman D, Rigamonti C, Shi A, Tumer Z, Van Balkom IDC, Hennekam RC (2018). Diagnosis and management of Cornelia de Lange syndrome: first international consensus statement. Nat Rev Genet.

[CR28] Kruszka P, Berger SI, Casa V, Dekker MR, Gaesser J, Weiss K, Martinez AF, Murdock DR, Louie RJ, Prijoles EJ, Lichty AW, Brouwer OF, Zonneveld-Huijssoon E, Stephan MJ, Hogue J, Hu P, Tanima-Nagai M, Everson JL, Prasad C, Cereda A, Iascone M, Schreiber A, Zurcher V, Corsten-Janssen N, Escobar L, Clegg NJ, Delgado MR, Hajirnis O, Balasubramanian M, Kayserili H, Deardorff M, Poot RA, Wendt KS, Lipinski RJ, Muenke M (2019). Cohesin complex-associated holoprosencephaly. Brain.

[CR29] Lee H, Deignan JL, Dorrani N, Strom SP, Kantarci S, Quintero-Rivera F, Das K, Toy T, Harry B, Yourshaw M, Fox M, Fogel BL, Martinez-Agosto JA, Wong DA, Chang VY, Shieh PB, Palmer CG, Dipple KM, Grody WW, Vilain E, Nelson SF (2014). Clinical exome sequencing for genetic identification of rare Mendelian disorders. JAMA.

[CR30] Li H, Ren Z, Kang X, Zhang L, Li X, Wang Y, Xue T, Shen Y, Liu Y (2009). Identification of tyrosine-phosphorylated proteins associated with metastasis and functional analysis of FER in human hepatocellular carcinoma cells. BMC Cancer.

[CR31] Maas SM, Shaw AC, Bikker H, Ludecke HJ, van der Tuin K, Badura-Stronka M, Belligni E, Biamino E, Bonati MT, Carvalho DR, Cobben J, de Man SA, Den Hollander NS, Di Donato N, Garavelli L, Gronborg S, Herkert JC, Hoogeboom AJ, Jamsheer A, Latos-Bielenska A, Maat-Kievit A, Magnani C, Marcelis C, Mathijssen IB, Nielsen M, Otten E, Ousager LB, Pilch J, Plomp A, Poke G, Poluha A, Posmyk R, Rieubland C, Silengo M, Simon M, Steichen E, Stumpel C, Szakszon K, Polonkai E, van den Ende J, van der Steen A, van Essen T, van Haeringen A, van Hagen JM, Verheij JB, Mannens MM, Hennekam RC (2015). Phenotype and genotype in 103 patients with tricho-rhino-phalangeal syndrome. Eur J Med Genet.

[CR32] Marcos-Alcalde I, Mendieta-Moreno JI, Puisac B, Gil-Rodriguez MC, Hernandez-Marcos M, Soler-Polo D, Ramos FJ, Ortega J, Pie J, Mendieta J, Gomez-Puertas P (2017). Two-step ATP-driven opening of cohesin head. Sci Rep.

[CR33] Martinez F, Caro-Llopis A, Rosello M, Oltra S, Mayo S, Monfort S, Orellana C (2017). High diagnostic yield of syndromic intellectual disability by targeted next-generation sequencing. J Med Genet.

[CR34] McBrien J, Crolla JA, Huang S, Kelleher J, Gleeson J, Lynch SA (2008). Further case of microdeletion of 8q24 with phenotype overlapping Langer-Giedion without TRPS1 deletion. Am J Med Genet A.

[CR35] McKay MJ, Troelstra C, van der Spek P, Kanaar R, Smit B, Hagemeijer A, Bootsma D, Hoeijmakers JH (1996). Sequence conservation of the rad21 *Schizosaccharomyces pombe* DNA double-strand break repair gene in human and mouse. Genomics.

[CR36] Mende RH, Drake DP, Olomi RM, Hamel BC (2012). Cornelia de Lange syndrome: a newborn with imperforate anus and a NIPBL mutation. Case Rep Genet.

[CR37] Minor A, Shinawi M, Hogue JS, Vineyard M, Hamlin DR, Tan C, Donato K, Wysinger L, Botes S, Das S, Del Gaudio D (2014). Two novel RAD21 mutations in patients with mild Cornelia de Lange syndrome-like presentation and report of the first familial case. Gene.

[CR38] Moss J, Penhallow J, Ansari M, Barton S, Bourn D, FitzPatrick DR, Goodship J, Hammond P, Roberts C, Welham A, Oliver C (2017). Genotype–phenotype correlations in Cornelia de Lange syndrome: behavioral characteristics and changes with age. Am J Med Genet A.

[CR39] Mulder PA, Huisman S, Landlust AM, Moss J, Consortium SA, Piening S, Hennekam RC, van Balkom IDC (2019). Development, behaviour and autism in individuals with SMC1A variants. J Child Psychol Psychiatry.

[CR40] Mullegama SV, Klein SD, Signer RH, Center UCG, Vilain E, Martinez-Agosto JA (2019). Mutations in STAG2 cause an X-linked cohesinopathy associated with undergrowth, developmental delay, and dysmorphia: Expanding the phenotype in males. Mol Genet Genomic Med.

[CR41] Mullenders J, Aranda-Orgilles B, Lhoumaud P, Keller M, Pae J, Wang K, Kayembe C, Rocha PP, Raviram R, Gong Y, Premsrirut PK, Tsirigos A, Bonneau R, Skok JA, Cimmino L, Hoehn D, Aifantis I (2015). Cohesin loss alters adult hematopoietic stem cell homeostasis, leading to myeloproliferative neoplasms. J Exp Med.

[CR42] Mungan Z, Akyuz F, Bugra Z, Yonall O, Ozturk S, Acar A, Cevikbas U (2003). Familial visceral myopathy with pseudo-obstruction, megaduodenum, Barrett’s esophagus, and cardiac abnormalities. Am J Gastroenterol.

[CR43] Newkirk DA, Chen YY, Chien R, Zeng W, Biesinger J, Flowers E, Kawauchi S, Santos R, Calof AL, Lander AD, Xie X, Yokomori K (2017). The effect of nipped-B-like (Nipbl) haploinsufficiency on genome-wide cohesin binding and target gene expression: modeling Cornelia de Lange syndrome. Clin Epigenet.

[CR44] Pati D, Zhang N, Plon SE (2002). Linking sister chromatid cohesion and apoptosis: role of Rad21. Mol Cell Biol.

[CR45] Pereza N, Severinski S, Ostojic S, Volk M, Maver A, Dekanic KB, Kapovic M, Peterlin B (2012). Third case of 8q23.3-q24.13 deletion in a patient with Langer-Giedion syndrome phenotype without TRPS1 gene deletion. Am J Med Genet A.

[CR46] Tsai CF, Wang YT, Yen HY, Tsou CC, Ku WC, Lin PY, Chen HY, Nesvizhskii AI, Ishihama Y, Chen YJ (2015). Large-scale determination of absolute phosphorylation stoichiometries in human cells by motif-targeting quantitative proteomics. Nat Commun.

[CR47] Watrin E, Kaiser FJ, Wendt KS (2016). Gene regulation and chromatin organization: relevance of cohesin mutations to human disease. Curr Opin Genet Dev.

[CR48] Woods SA, Robinson HB, Kohler LJ, Agamanolis D, Sterbenz G, Khalifa M (2014). Exome sequencing identifies a novel EP300 frame shift mutation in a patient with features that overlap Cornelia de Lange syndrome. Am J Med Genet A.

[CR49] Wuyts W, Roland D, Ludecke HJ, Wauters J, Foulon M, Van Hul W, Van Maldergem L (2002). Multiple exostoses, mental retardation, hypertrichosis, and brain abnormalities in a boy with a de novo 8q24 submicroscopic interstitial deletion. Am J Med Genet.

[CR50] Yuan B, Neira J, Pehlivan D, Santiago-Sim T, Song X, Rosenfeld J, Posey JE, Patel V, Jin W, Adam MP, Baple EL, Dean J, Fong CT, Hickey SE, Hudgins L, Leon E, Madan-Khetarpal S, Rawlins L, Rustad CF, Stray-Pedersen A, Tveten K, Wenger O, Diaz J, Jenkins L, Martin L, McGuire M, Pietryga M, Ramsdell L, Slattery L, Study DDD, Abid F, Bertuch AA, Grange D, Immken L, Schaaf CP, Van Esch H, Bi W, Cheung SW, Breman AM, Smith JL, Shaw C, Crosby AH, Eng C, Yang Y, Lupski JR, Xiao R, Liu P (2019). Clinical exome sequencing reveals locus heterogeneity and phenotypic variability of cohesinopathies. Genet Med.

[CR51] Yuen RK, Thiruvahindrapuram B, Merico D, Walker S, Tammimies K, Hoang N, Chrysler C, Nalpathamkalam T, Pellecchia G, Liu Y, Gazzellone MJ, D’Abate L, Deneault E, Howe JL, Liu RS, Thompson A, Zarrei M, Uddin M, Marshall CR, Ring RH, Zwaigenbaum L, Ray PN, Weksberg R, Carter MT, Fernandez BA, Roberts W, Szatmari P, Scherer SW (2015). Whole-genome sequencing of quartet families with autism spectrum disorder. Nat Med.

[CR52] Zhang BN, Chan TCY, Tam POS, Liu Y, Pang CP, Jhanji V, Chen LJ, Chu WK (2019). A cohesin subunit variant identified from a peripheral sclerocornea pedigree. Dis Mark.

