# Supplementary material for: Delineation of phenotypes and genotypes related to cohesin structural protein RAD21
Source: Hum Genet. 2020 Mar 19;139(5):575–92. doi: 10.1007/s00439-020-02138-2 (PMC7170815; doi:10.1007/s00439-020-02138-2)
Supplement: Supplementary file 1 — Supplementary material 1 (PDF 1941 kb) [file 439_2020_2138_MOESM1_ESM.pdf]

Supporting Information to

Delineation of phenotypes and genotypes related to cohesin structural protein RAD21

Lianne C. Krab, Iñigo Marcos-Alcalde, Melissa Assaf, Meena Balasubramanian, Janne Bayer Andersen, Anne-Marie Bisgaard Pedersen, David R. Fitzpatrick , Sanna Gudmundsson, Sylvia A. Huisman, Tugba Kalayci, Saskia M. Maas, Francisco Martínez, Shane McKee, Leonie A. Menke, Paul A. Mulder, Oliver D. Murch, Michael Parker, Juan Pie, Feliciano J. Ramos, Claudine Rieubland, Jill A. Rosenfeld Mokry, Emanuela Scarano, Marwan Shinawi, Paulino Gómez-Puertas, Zeynep Tümer, Raoul C. Hennekam

Contents:

A: Supporting Information methods:

Patient selection, Structure modeling of RAD21 variants, Molecular dynamics simulations.

B: Supporting Information figures

Supporting figure S1: Scheme of the cohesin ring showing location of the domains of the protein RAD21 bound to SMC3 (RAD21-SMC3), to SMC1A (RAD21-SMC1A) and to STAG1/2 protein.

Supporting figure S2. Structural modeling of RAD21-N terminal (RAD21-SMC3) domain in complex with the SMC3 head harboring the variant Arg65Gln.

Supporting figure S3. Structural modeling of RAD21-STAG domain in complex with STAG2 harboring the variants Ser345Pro, and Pro376Arg.

Supporting figure S4: additional pictures of clinical phenotype of *RAD21* patients in cohort A

Supporting materials C: Supporting tables

Supporting table S1: Patient selection

Supporting table S2: CdLS clinical scores in cohort A (n=30).

Supporting table S3: individual clinical characteristics in patient cohort A (n=30) based on report on dedicated questionnaire, or additional anomalies reported spontaneously.

Supporting table S4: Individual data on cognitive functioning, behavior and developmental milestones in cohort A (n=30)

Supporting table S5: available clinical information for Cohort B (n=37 cases)

Supporting table S6: Patients not included in this study with unusual features

Supporting materials D: Literature references with supporting information

A: Supporting methods

Patient selection

We used four strategies to acquire affected individuals. First, we performed a PubMed search using as search terms ‘Cornelia de Lange Syndrome AND *RAD21*’, ‘*RAD21*’ (selection: case reports or reviews)’, ‘RAD21 AND clinical’ or ‘8q24.1’ (selection: case reports or reviews), which yielded 62, 38, 86 and 23 hits, respectively (date: 06-15-2019). We screened title and abstract for the terms RAD21 and 8q24.1, and hand searched reference lists to collect additional publications. After applying exclusion criteria (see below) we contacted authors of suitable papers and asked for updated clinical information. Second, we contacted the 43 participants of the international CdLS Consensus Group (Kline et al., 2018) and the academic clinical genetic laboratories in the Netherlands for unpublished individuals with *RAD21* variants. Third, we performed database searches in Decipher ("Decipher,") and ClinVar ("ClinVar,") for unpublished Copy Number Variations (CNVs) and sequence variants involving *RAD21*. After applying exclusion criteria (see below), we invited colleagues who had entered suitable candidates in Decipher to contribute to the present series. For ClinVar, anticipating a low response rate, we only contacted the laboratories and clinicians for additional clinical data if variants were in the RAD21 protein binding domains. Lastly, we invited colleagues who had contacted the senior author (RCH) for management advice in an individual with a *RAD21* variant, to participate.

We removed duplicates, and excluded cases if 1. No clinical data was available; 2. The variant was reportedly benign, likely benign or equivalent (variants reported as a variant of uncertain significance (VUS) were eligible for further analysis); 3. After further analyses variants were (still) a VUS or turned out to be (likely) benign; 4. CNV breakpoints were not specified; 5. There were CNVs elsewhere in the genome, or intragenic variants in other genes or 6. CNVs encompassed regional genes that may influence the *RAD21* phenotype. Thus, we limited CNVs to a maximum size of 3.0Mb, which downstream did not overlap with *TRPS1* and upstream did not overlap with *SAMD12* (in which repeat expansion is associated with adult myoclonia, but effects of a (partial) deletion could not be ruled out (Coffee et al., 2008) (see figure 1). We did not exclude deletions encompassing morbid genes *SLC30A8* and *EXT1*, as *SLC30A8* is associated with noninsulin dependent diabetes mellitus (late onset) only, and the phenotype linked to *EXT1* is limited to exostoses and other specific bone anomalies which can easily be discerned from the *RAD21* phenotype. Also, we did not select against deletions overlapping non-morbid genes in the region between *TRPS1* and *SAMD12* (*EIF3H, UTP23, AARD,* and *MED30*) as these are not currently known to be associated with a clinical phenotype.

Structure modeling of RAD21 variants

A set of three structure models for different domains of wild-type human RAD21 protein in complex with their accompanying proteins was generated: The protein domains which can be modelled are: N-terminal domain (RAD21-SMC3, residues 18-87, containing part of the SMC3 binding domain that spans residues 1-103), the region around the STAG1/2 binding domain (RAD21-STAG, residues 321-392; STAG1/2 bind to residues 362–403) and C-terminal domain (RAD21-SMC1A, residues 543-628; containing the SMC1A binding domain on residues 558–628). The model for RAD21-SMC3 was obtained using the structure of the cohesin Smc3-HD:Scc1-N complex from yeast as template (PDB id: 4UX3; (Gligoris et al., 2014)). The template used to build the RAD21-STAG model was the crystal structure of human Stromal Antigen 2 -SA2- in complex with Sister Chromatid Cohesion protein 1 -Scc1- (PDB id: 4PJU; (Hara et al., 2014)). In the case of the RAD21-SMC1A domain, a model previously published for the trimeric complex of human SMC1A-head, SMC3-head and RAD21-SMC1A domains was used (Gudmundsson et al., 2018; Marcos-Alcalde et al., 2017). With these three wild-type models as templates, 12 *RAD21* missense variants were modeled. All protein complex models were built using the SWISS-MODEL server (http://swissmodel.expasy.org), their structural quality being within the accepted range for homology-based structure (Anolea/Gromos/ QMEAN4; (Benkert, Biasini, & Schwede, 2011)).

Molecular Dynamics simulations

To analyze the putative effect of the variants on the RAD21 structure, the behavior of the 15 models (twelve variant proteins and the corresponding three wild type models) were compared by subjecting all to a free molecular dynamics (MD) simulation using the AMBER18 molecular dynamics package (http://ambermd.org/; University of California-San Francisco, CA). Duration of the MD exposure was 60 nanoseconds (ns) for RAD21-SMC3 and RAD21-STAG, and or 100 ns for RAD21-SMC1A (based on the large size of the trimeric structure of the RAD-SMC1A system and its internal complexity, 40 ns were added to ensure that conformational stability was reached for each trajectory; see the wild-type trajectory in figure 2). In all cases, the 3D models were solvated with a periodic octahedral pre-equilibrated solvent box using the LEaP module of AMBER, with 12 Ångstroms (Å) as the shortest distance between any atom in the protein complex and the periodic box boundaries. The PMEMD program of AMBER18 and the ff14SB force field (http://ambermd.org/) were used to perform the free MD simulations, applying the SHAKE algorithm, a time step of 2 femtoseconds (fs), and a non-bonded cut-off of 12Å. Initial relaxation of the systems was done over 10,000 steps of energy minimization, using 1,000 steps of steepest descent minimization followed by 9,000 steps of conjugate-gradient minimization. In the course of the subsequent 20 picoseconds (ps) long heating phase, the temperature was raised from 0 to 300 K in 10 change steps, after each of which velocities were reassigned. This phase was followed by a equilibration phase in which the initial force constant for Cα trace dihedrals

restriction was progressively reduced from 500 to 0 kcal mol^{-1} rad^{-2} over 200 ps. After this equilibration phase, 60 or 100 ns of unrestricted MD simulation were obtained for each system. MD trajectories were analyzed using VMD software (Humphrey, Dalke, & Schulten, 1996). The movements in the structure of the models during the trajectories were continuously monitored by the measurement of root-mean square deviation (RMSD) of atomic positions. In brief, large variations of RMSD values indicate notable distortions of the protein structure probably due to the presence of the new amino acid (variant) in the structure when compared to the wild type protein. Figures were generated using the Pymol Molecular Graphics System (https://pymol.org/; Schrödinger, LLC, Portland, OR).

Supporting materials B: Supporting figures

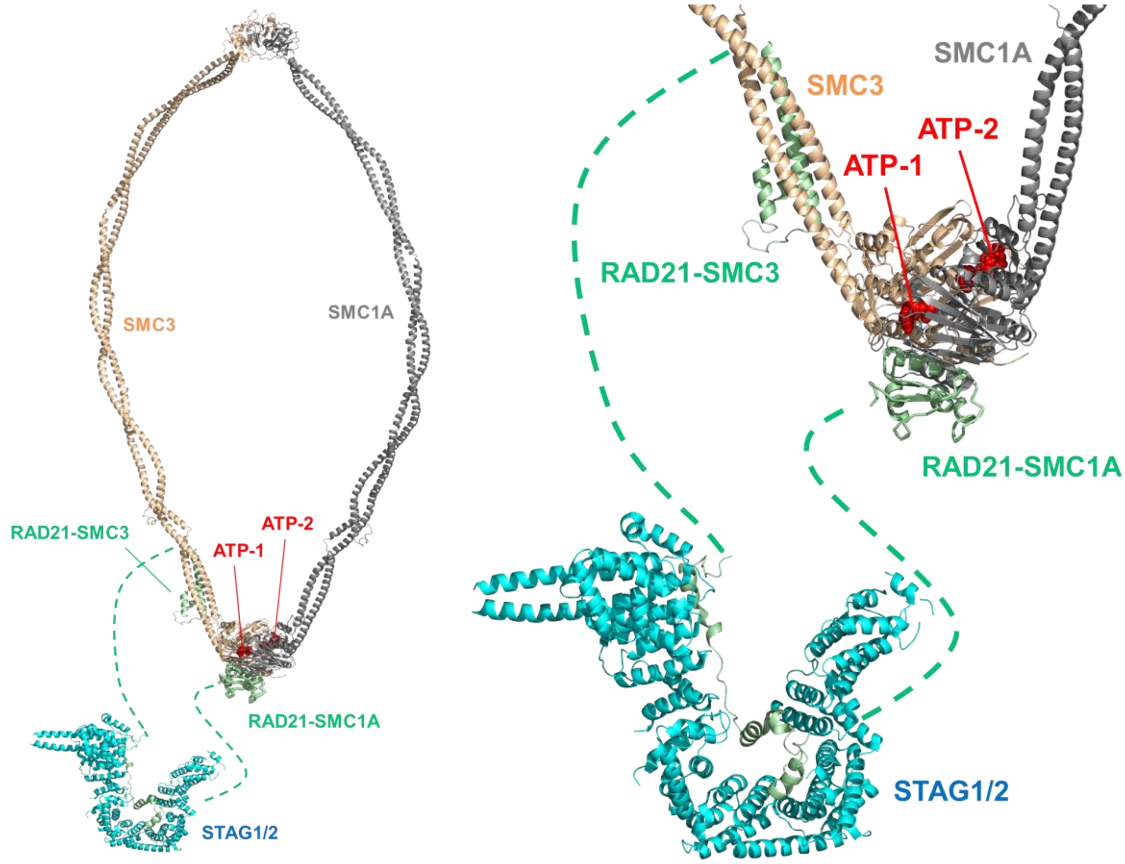

Supporting Fig. S1: Scheme of the cohesin ring showing location of the domains of the protein RAD21 bound to SMC3 (RAD21-SMC3), to SMC1A (RAD21-SMC1A) and to STAG1/2 protein.

Unstructured, not-modeled domains of RAD21 protein are represented as dashed lines. The position of two ATP molecules located between the head domains of SMC1A and SMC3 is indicated (red spheres). The SMC3/SMC1A ring structure was modeled using the *Pyrococcus yayanosii* SMC ring structure (Diebold-Durand et al., 2017) as template, courtesy of Hansol Lee, Byung-Ha Oh and Stephan Gruber.

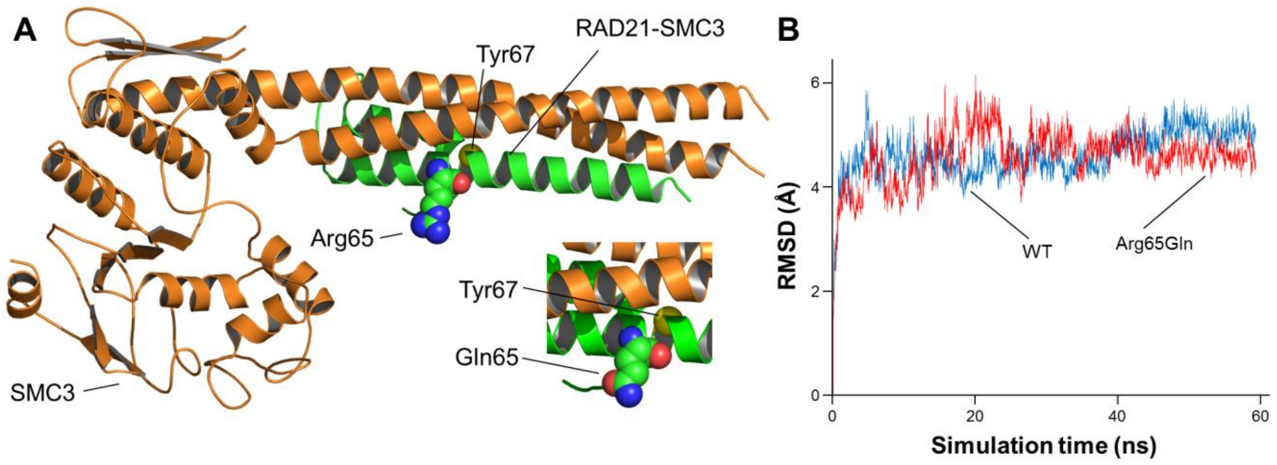

Supporting Fig. S2 A-B. Structural modeling of RAD21-N terminal (RAD21-SMC3) domain in complex with the SMC3 head harboring the variant Arg65Gln.

A. Model for the RAD21-SMC3 structured sub-domain (residues 18-87, green). The RAD21-SMC3 structure contains two alpha helices, the second one being tightly coupled with the coiled-coil moiety of the SMC3 head domain (orange). Positions of the wild-type (Arg65) and mutated (Gln65) amino acids are indicated in the upper and lower figure, respectively. The Arg65 residue is located in this second alpha helix, oriented towards the solvent, in the opposite direction to the coiled-coil, in close proximity to Tyr67. Neither Arg65 nor Gln65 has an apparent contact to the SMC3 coiled-coil structure.

B. Root mean square deviation of the atomic positions (RMSD, in Angstroms (Å)) of modeled structures (WT: wild-type, blue line; Arg65Gln variant, red line) during 60 ns of free molecular dynamics simulation. The graphic shows that there are no major differences in RMSD values when comparing the trajectories.

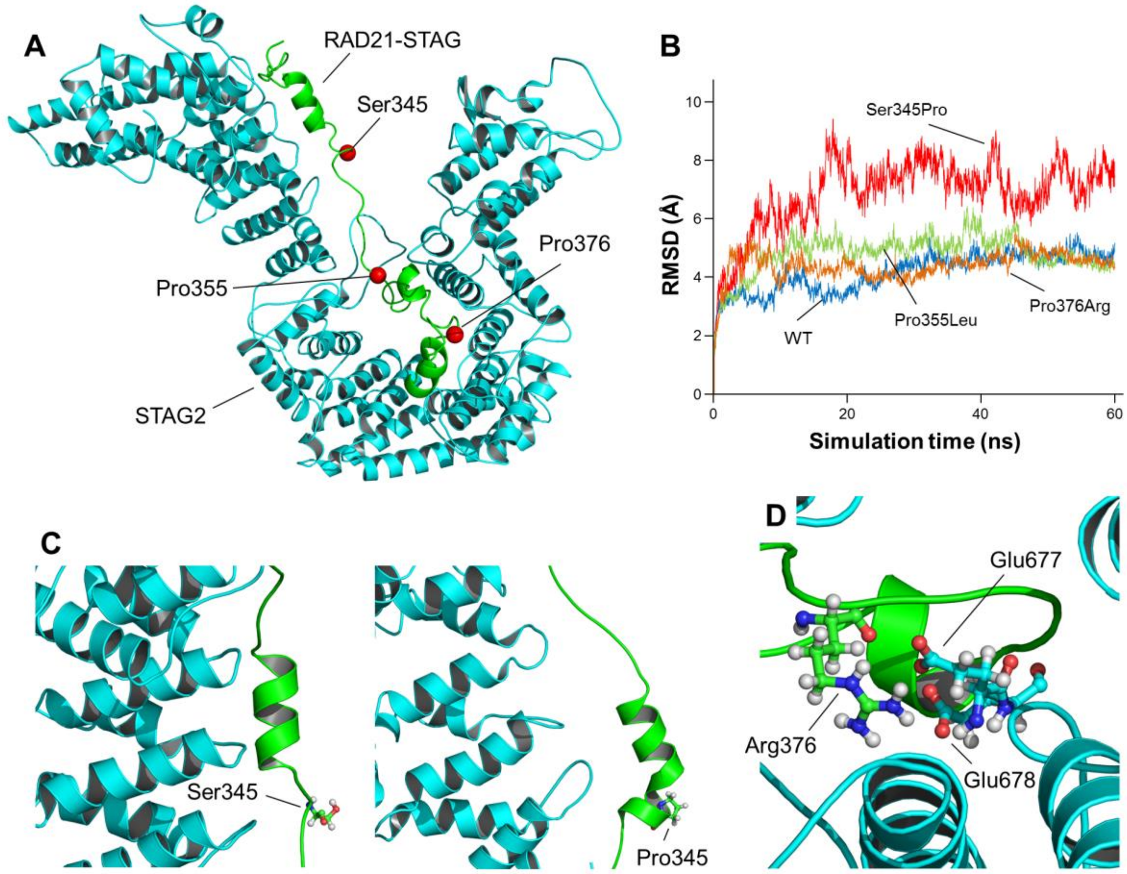

Supporting Fig. S3 A-D. Structural modeling of RAD21-STAG domain in complex with STAG2 harboring the variants Ser345Pro, and Pro376Arg.

Supporting Fig. S3 A-D continued

A. Model for RAD21 residues 321-392 (green) bound to the surface of STAG2 protein (cyan). The positions of the mutated residues Ser345 and Pro376, as well as Pro355 (see Supplementary data S6), are indicated with red spheres.. The RAD21-STAG1/2 domain is involved in binding of STAG1/2. Modelling indicates that it is arranged in three small alpha helices distributed along a mainly non-structured coil. These were adjacent to the inner face of the STAG2 protein, which is conformed in a HEAT-repeat structure (Fig protein 2A). Mutated residues Ser345 and Pro376 are located in the non-structured segments of the RAD21-STAG.

B. Root mean square deviation (RMSD, in Angstroms (Å)) of modeled structures (WT: wild-type, blue line; Ser345Pro, red line; Pro355Leu, green line (see Supplementary data S6); Pro376Arg, orange line) during 60 ns of free Molecular Dynamics stimulation. The structure of the variant Ser345Pro exhibits a notable variation, reaching RMSD values of more than 8 Å frequently along the trajectory. In contrast, RMSD values trajectory of the mutant protein Pro376Arg show little differences compared to the wild-type.

C. Structure of RAD21 around the position 345 in WT (left) and variant Ser345Pro (right) models after 60 ns of MD, showing the notable distortion and movement of the subdomain in presence of the variant. Fitting with the large variation in RMSD values, after the first 10 ns of MD, the variant Ser345Pro promotes the *de novo* generation of a curved small alpha helix segment, bound to the pre-existing alpha helix that separates from the surface of the STAG2 protein. This arrangement remains constant until the end of the 60 ns trajectory

D. Position of the mutated residue Pro376 (Pro376Arg) in stable contact with the residues Glu677 and Glu678 located on the surface of STAG2 after 60 ns of MD. In consonance with their lack of RMSD variation, no clear structural movements can be observed for Pro376Arg on RAD21 itself, but the Pro376Arg variant protein still affects the RAD21-STAG2 interaction, as a salt bridge is formed between the mutated positively charged Arg376 residue and the negatively charged STAG2 residues Glu677 and Glu678. This strong interaction is maintained along the entire 60 ns trajectory, which is predicted to cause over-stabilization of the interaction between the two proteins.

Supporting Fig. S4 A-C: Additional pictures of clinical phenotype of *RAD21* patients in cohort A

F: Family, y: years, m: months. Family numbers of individuals in the panels correspond to family numbers in the tables. Ages are mentioned below each picture. For detailed descriptions please see Tables and text.

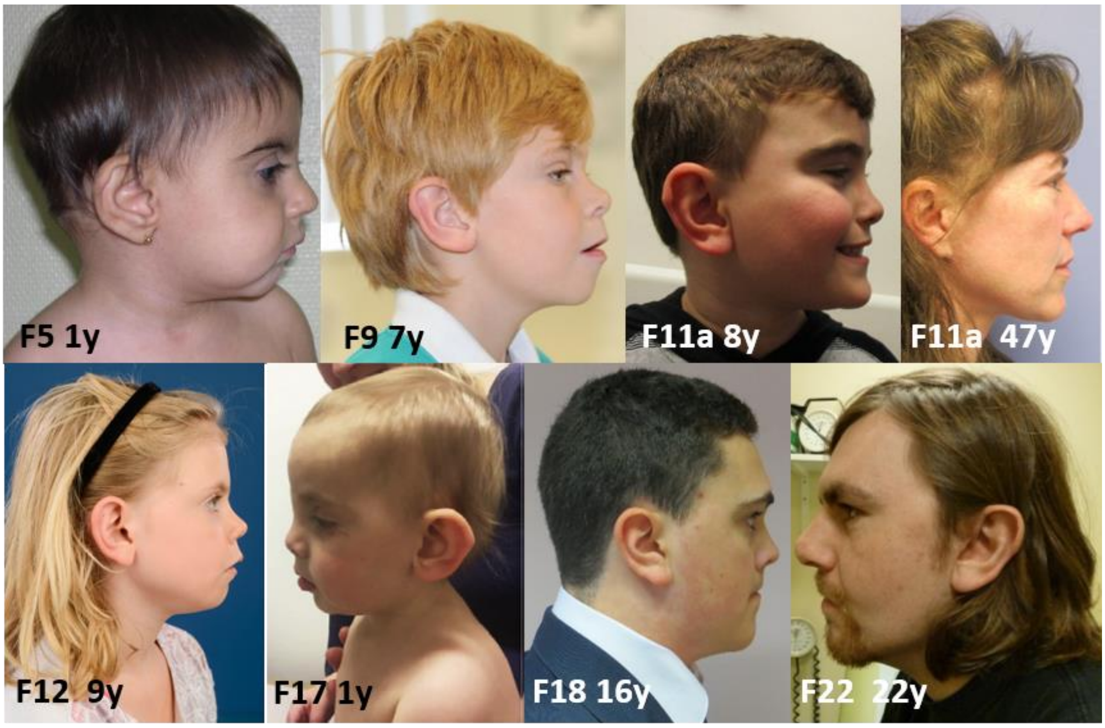

Fig. 4a. Side facial view of a limited number of presently reported individuals

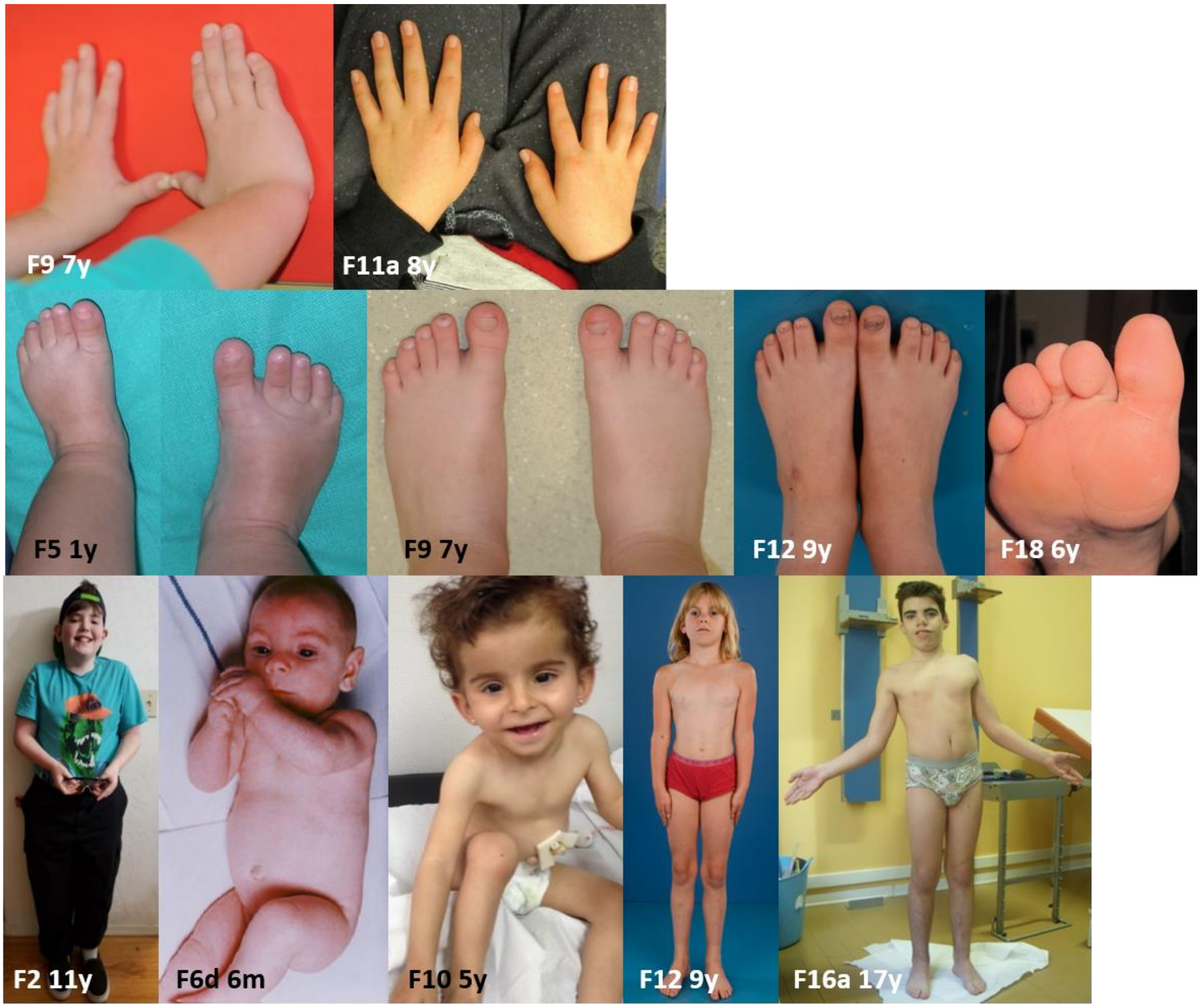

Fig. 4B. Distal limb characteristics and general body build in a limited number of presently reported individuals.

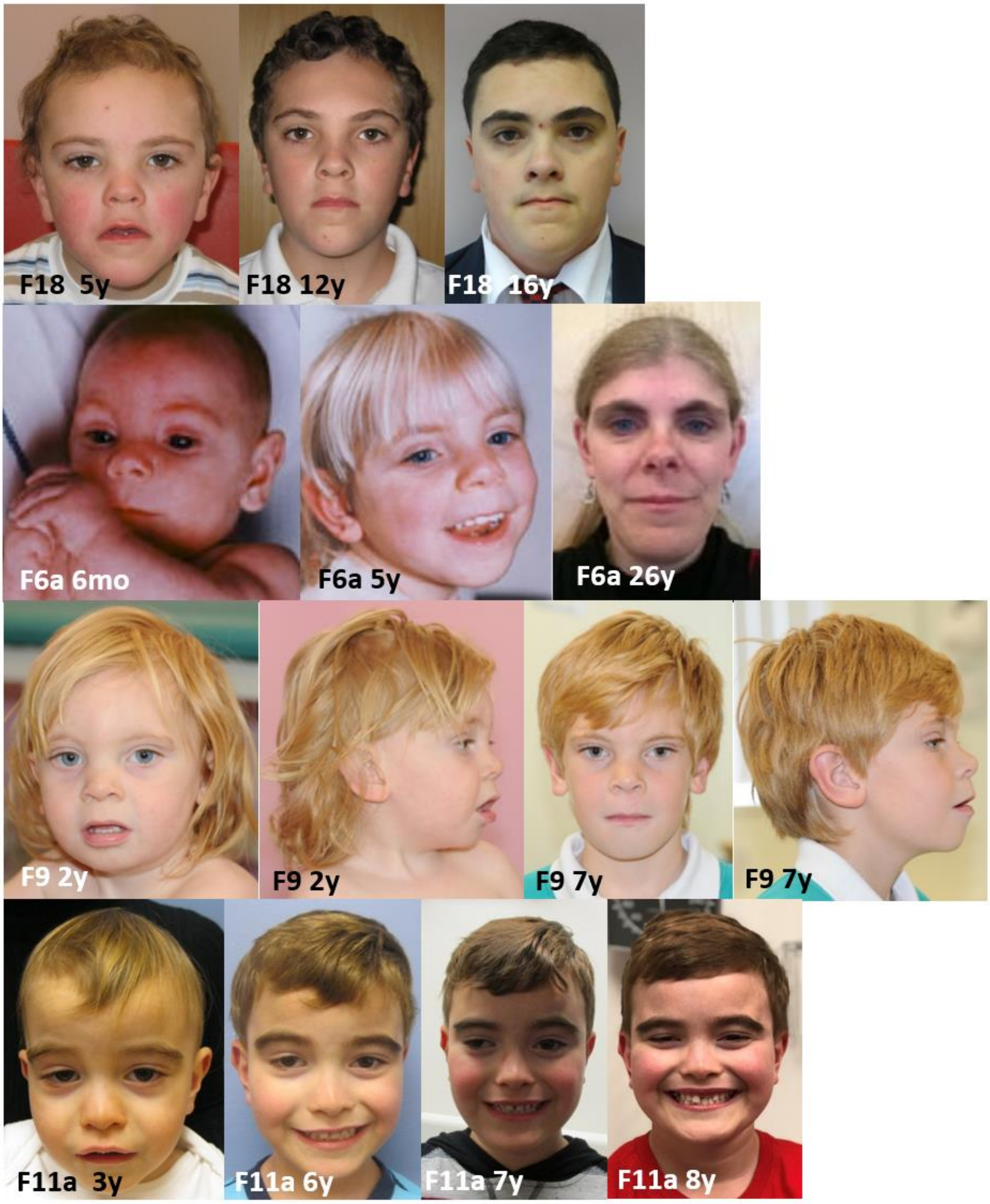

Fig. 4C. Changes with age in a limited number of presently reported individuals.

Supporting materials C: Supporting tables

Supporting Table S1: Patient selection

Source	Number + type	Exclusion	Included in cohort A	Included in cohort B	Included total
Decipher	11 seq var	- Duplicates (4)<br>- other variants (2)<br>- no clinical data upon request (1)	0	4 seq var (3 fam)	4 seq var (3 fam)
Decipher	17 del<br>9 dup	- Too large (9 del, 2 dup)<br>- Other CNVs (1 del)<br>- Combination (3 del, 7 dup)<br>- No clinical data upon request (4 del)	0	0	0
ClinVar	54 seq var	- duplicates (8)<br>- Other variants (2)<br>- (likely) benign (25, of which 1 in retrospect after enquiry)<br>- VUS remained a VUS or was (likely) benign after analyses (8)<br>- No clinical data (10, of which 1 upon request)	1 seq var (1 fam)	0	1 seq var (1 fam)
ClinVar	18 del<br>36 dup	- Duplicates (4 del, 4 dup)<br>- Too large (9 del, 29 dup)<br>- (likely) benign (2 del)<br>- VUS was likely benign after analyses (1 dup)<br>- No clinical data (1 dup)	0	3 del (3 fam)	3 del (3 fam)
Literature unique articles	34 seq var	- Duplicate (2)<br>- Other variants (1)<br>- Relationship of phenotype to RAD21 unclear; VUS (8)	14 seq var (10 families)	9 seq var<br>(4 families + 1 FCA)	23 seq var<br>(14 families)
Literature unique articles	20 del	- Too large (18 del)	2 del (2 families)	0	2 del (2 families)
Colleagues	14 seq var	0	10 seq var (7 fam + 1 FCA)	4 seq var (1 fam + 1 FCB)	14 seq var (8 fam + 2 FCA/FCB)
Colleagues	3 del<br>3 dup	- Too large (1 del)<br>- VUS was likely benign after analyses (1 dup)<br>- No clinical phenotype (2 dup)	2 del (2 families)	0	2 del (2 fam)
**Total**	**219 cases**	**169 cases<br>(of which 22 duplicates)**	**29 cases from 22 families<br>(25 seq var, 4 del)**	**20 cases from 11 families<br>(17 seq var, 3 del)**	**49 cases from 34 families<br>(42 seq var, 7 del)**

CNV: copy number variants. Del: microdeletions, dup: microduplications, seq var: sequence variants, VUS: variant of uncertain significance, sequence: sequence variants, Fam: families, FCA/FCB: families already in cohort A/B

Supporting Table S2: CdLS clinical scores in cohort A (n=29).

Patient ID	F1	F2	F3a	F4	F5	F6a	F6b	F6c	F6d	F6e	F7	F8	F9	F10	F11a	F11b
Age (years;months)	5	11	3;3	12	1	26	61	60	50	44	5	6	23	4	7;6	46
Gender	M	M	F	M	F	F	F	F	F	F	M	M	M	F	M	F
Cardinal features (2 points each if present)																
Synophrys and/or thick eyebrows	+	+	+	+	+	+	+	+	+	+	+	+	+	+	+	+
Synophrys	-	+	+	+	+	+	+	+	+	+	+	+	+	-	+	+
Thick eyebrows	+	+	-	+	-							+	+	+	+	+
Short nose, concave nasal ridge, and/or upturned nasal tip	+	+	+	+	+	+	+	+	+	+	+	+	+	+	+	-
Short nose	+	+	+	+	+	+	+	+	+	+		+	+	+	-	-
Concave nasal ridge	-	-	+	-	+	-	-	-	-	-	+	+	+	-	+	-
Upturned nasal tip	-	-	+	+	-	+	+	+	+	+	+	+	+	-	-	-
Long and/or smooth philtrum	+	-	+	+	+	+	+	+	+	+	+	+	+	+	+	-
Long philtrum	+	-	-	+	+	+	-	+	+	+	+	+	+	+	-	-
Smooth philtrum	-	-	+	+	-	+	+	-	+	+		+		+	+	-
Thin upper lip vermilion and/or Downturned corners of mouth	-	-	+	+	-	+	+	+	+	+	+	+	+	+	-	+
Thin upper vermillion	-	-	+	+	-	+	+	+	+	+	+	+	+	+	-	+
Thin lips, downturned corners	-	-	+	-	-	+	+	+	+	+	+	+	+	+	-	-
Hand oligodactyly and/or adactyly†	-	-	-	-	-	-	-	-	-	-	-	-	-	-	-	-
Diaphragmatic hernia†	-	-	-	-	-	-	-	-	-	-	-	-	-	-	-	-
Suggestive features (1 point each if present)																
Global DD and/or ID‡	+	+	+	+	+	+	+	-	-	+	+	+	+	+	+	-
Prenatal growth retardation§	-		-	-	-	-	-		-	-	-		-	-	-	-
Postnatal growth retardation¶	+	-	-	-	-	+	-	-	-	-	-	-	-	-	-	-
Microcephaly prenatally or postnatally	post +	post-	pre -<br>post +	post +	pre +<br>post -	post +	-	-	post +	-	pre -<br>post +	post +	pre -<br>post +	-	post -	post -
Small hands and/or feet	-	-		-	-	-	-	-	-	+			+	-	-	-
Short 5th finger††	+	+		+	-	+	+	+	-	+	+		+	+	+	+
Clinodactyly 5th finger		+		+	-	+	+	+	-	-	+		+	+	+	-
Hirsutism	-	+		+	+	-	-	-	-	+	-		+	-	-	-
CdLS score‡‡	9	≥7	≥10	12	9	12	10	≥9	9	12	≥11	≥10	13	10	8	5
Diagnosis based on score	NC		NC	C	NC	C	NC	NC	NC	C	C	NC§§	C	NC	-	-
CdLS suspected prior to molecular testing	-	-	+	+	-	+	-	-	-	-	+	+; NC	+	+	+	-

Patient ID	F12	F13	F14a	F14b	F15	F16a	F16b	F17	F18	F19	F20	F21	F22
Age (years;months)	9	'child'	5;11	0	13	17	46	2	6;5	'child'	2;1	5	22
Gender	F	F	F	F	M	M	M	M	M	M	F	M	M
Cardinal features (2 points each if present)													
Synophrys and/or thick eyebrows	+	+	+	+	+	+	-	+	+	+	+	+	+
Synophrys	-	+	+	+	-	+	-	-	+	-		-	+
Thick eyebrows	+	+	+	-	+	+	-	+	+	+	+	+	+
Short nose, concave nasal ridge, and/or upturned nasal tip	+	+	+	+	+	+	+	+	+	+	+	+	+
Short nose	+	+	+	+	+	-	+	+	+			+	+
Concave nasal ridge	-	+	-	+	-	+	+	-	-	+	+	+	-
Upturned nasal tip	+		+	+	+	+	+	+	+		+	-	-
Long and/or smooth philtrum	+	+	+	+	+	+	+	-	+	+	+	+	+
Long philtrum	+	+	+	+	+	+	+	-	+	+	-	+	+
Smooth philtrum	+	+	+	+	-	+	-	-	+		+		+
Thin upper lip vermilion and/or Downturned corners of mouth	+	+	+	+	+	-	+	-	+	+	+	+	+
Thin upper vermillion	+	+	+	+	+	-	+	-	+	+	+	+	+
Thin lips, downturned corners	-	+	+	+	+	-	+	-	+			-	-
Hand oligodactyly and/or adactyly†	-	-	-	-	-	-	-	-	-	-	-	-	-
Diaphragmatic hernia†	-	-	-	-	-	-	-	+	-	-	-	-	-
Global DD and/or ID‡	+	+	+	n/a	+	+	+	+	+	+	n/a	+	+
Prenatal growth retardation§	+		-	+		-	-	-	-		+	+	-
Postnatal growth retardation¶	+		-	n/a	+	+	+	-	-	+	+	+	+
Microcephaly prenatally or postnatally	pre + post +	post +	-	pre +	post +	-		pre + post +	post -	post +	pre + post +	pre + post +	pre + post +
Small hands and/or feet	-	+	+	-	+	-	-	-	-	-	-	-	-
Short 5th finger††	+	+	+	-		+	+	-	-				+
Clinodactyly 5th finger	-	+	-	-		+	+	-	-	-	-		+
Hirsutism	-	-	+	-		+	+	-	-	+	+	-	-
CdLS score‡‡	13	≥12	12	≥10	≥12	10	≥10	8	9	≥12	≥12	≥12	12
Diagnosis based on score	C	C	C	NC§§	C	NC	NC§§	-	NC	C	C	C	C
CdLS suspected prior to molecular testing?	-	+; NC	+	+	+	-; KBGS	-	-	-	+?	?	-; LGS	-

+: present, -: not present, ≥: at least; due to missings, n/a: not available due to age; DD: global developmental delay (delay in at least the 2 domains of development speech or motor functioning), LD: learning disability, ID: intellectual disability, mod: moderate, sev: severe, CdLS: Cornelia de Lange Syndrome, C: Classic CdLS, NC: Non-Classic CdLS, KBGS; KBG syndrome, LGS: Langer-Giedion Syndrome.

†If not specifically mentioned as being present, we assumed negative and scored negative.

‡for the purpose of CdLS score: scored positive if ID or DD was present at any age (differs from definition in table 3 and S5)

§Birth weight <-2SD

Supporting Table S2 continued

¶Height <-2SD

††Short 5th finger is scored positive if reported or if clinodactyly is present and missing if not reported or if clinodactyly is negative, as clinodactyly invariably leads to a short 5th digit in CdLS, but short 5th finger can be present in the absence of clinodactyly.

‡‡Based on Kline et al 2018; Score <4 is insufficient to indicate molecular testing for CdLS, 4-8 indicates molecular testing for CdLS; score 9-10 is classified as non-classic CdLS; score 11 or higher is classified as classic CdLS. the '≥' sign in front of the CdLS score indicates 'at least', if clinical features were missing. If this potentially influenced the classification into CdLS categories, this is indicated with "§§"

§§If missing data were positive, total score could indicate Classic CdLS

Supporting Table S3: Individual clinical characteristics in patient cohort A (n=30) based on report on dedicated questionnaire, or additional anomalies reported spontaneously.

Anomaly in Dedicated Questionnaire	Affected individual
Length at birth <-2SD	F20, F21
Weight at birth <-2SD	F12, F14b, F20, F21
Prenatal head circumference <-2SD	F5, F7, F11, F14b, F20, F21, F22
Postnatal height <-2SD	F6a, F12, F15, F16a, F16b, F19, F21, F22
Postnatal weight <-2SD	F1, F17, F20
Postnatal head circumference <-2SD	F1, F3a, F4, F6a, F6d, F7, F8, F9, F12, F13, F15, F17, F19, F20, F21, F22
Brachycephaly	F4, F5, F6b, F6d, F6e F9, F10, F18
Low anterior/posterior hairline	F2, F4, F5, F6a, F6b, F6c, F6d, F6e, F9, F14a, F16a, F16b, F20, F22
Arched eyebrows	F1, F5, F6a, F6b, F6c, F6d, F6e, F8, F9, F11a, F11b, F12, F13, F14b, F15, F16a, F16b, F17, F20, F21, F22
Synophrys	F2, F3a, F4, F5, F6a, F6b, F6c, F6d, F6e,F7, F8, F9, F11a, F11b, F13, F14a, F14b, F16a, F18, F22
Thick eyebrows	F1, F2, F4, F6e, F8, F9, F10, F11a, F11b, F12, F13, F14a, F15, F16a, F17, F18, F19, F20, F21, F22
Long eyelashes	F2, F4, F5, F6a, F6e, F7, F8, F9, F10, F11a, F11b, F13, F14a, F14b, F15, F16a, F17, F19, F20, F21, F22
Concave nasal ridge	F3a, F5, F6e, F7, F8, F9, F11a, F13, F14b, F16a, F16b, F19, F20, F21
Upturned nasal tip	F3a, F4, F6a, F6b, F6c, F6d, F6e, F7, F8, F9, F12, F14a, F14b, F15, F16a, F16b, F17, F18, F20
Short nose	F1, F2, F3a, F4, F5, F6a, F6b, F6c, F6d, F6e, F8, F9, F10, F12, F13, F14a, F14b, F15, F16b, F17, F18, F21, F22
Long and/or smooth philtrum	F1, F3a, F4, F5, F6a, F6b, F6c, F6d, F6e, F7, F8, F9, F10, F11a, F12, F13, F14a, F14b, F15, F16a, F16b, F18, F19, F20, F21, F22
Thin upper lip vermillion	F3a, F4, F6a, F6b, F6c, F6d, F6e, F7, F8, F9, F10, F11b, F12, F13, F14a, F14b, F15, F16b, F18, F19, F20, F21, F22
Thin lips, downturned corners mouth	F3a, F6a, F6b, F6c, F6d, F6e, F7, F8, F9, F10, F13, F14a, F14b, F15, F16b, F18, F22

Highly arched palate	F6e, F9, F10, F14b, F16a, F16b, F17, F18
Cleft palate or submucous cleft palate	F6c, F8, F9, F12 (part of Pierre Robin sequence), F16b, F18
Widely spaced or absent teeth	F9, F11a
Micrognathia	F4, F5, F9, F11a, F11b, F12, F14b, F17,
Low-set and/or malformed ears	F1, F2, F4, F5, F6a, F6b, F6c, F6d, F9, F16a, F16b, F17, F20, F22; if specified: large and protruding (9/9).
Major limb malformation	-
Small hands	F6e, F9, F13, F14a, F15
Proximally placed thumb	F6a, F6d, F9, F10, F14a, F19,
Clinodactyly 5th finger	F2, F4, F6a, F6b, F6c, F7, F9, F10, F11a, F13, F16a, F16b, F22
Short 5th finger	F1, F2, F4, F6a, F6b, F6c, F6e, F7, F9, F10, F11a, F11b, F12, F13, F14a, F16a, F16b, F22
Syndactyly fingers	F4
Abnormal palmar crease	F4, F8, F14a, F14b, F16a, F16b, F17, F21, F22
Dislocated elbow/abnormal extension	F5, F6a, F6b, F6c, F6d, F6e, F8, F10, F12, F16a, F16b,
Small feet	F9, F13, F15
Syndactyly 2nd-3rd toes	F4, F8, F13, F22
Pectus excavatum	F4, F5, F16a
Pectus carinatum	F8
Scoliosis	F16a (with hemivertebrae), F16b
Hip dysplasia	Coxa vara and short femoral neck (F8); unspecified (F10), both below age 7.
Ptosis	F3a, F4, F5, F6a, F8, F11a, F15, F16a, F17, F20, F21
Visual impairment	-
Myopia ≥ -6.00 D	'myopia' (F1, F7), 'mild myopia' (F4, F11b)
Hearing loss	Mild conductive, resolved (F11a), Mild (F6c, F16b), Mild, high frequency (F22), Moderate mixed (F5), Moderate (F6a), hearing loss, not specified, due to staples fixation (F8), Hearing loss, not specified (F6b)
Seizures	F2 (refractory from age 6y), F22 (between ages 3 and 7)
Cutis marmorata	F7, F13, F21
Hirsutism	F2, F4, F5, F6e, F9, F14a, F16a, F16b, F19, F20
CNS major and minor malformations	Septo-optic dysplasia (F9), arachnoid cyst (F16a)

Heart (major and minor)	'Mild structural heart disease' (F6c), Tetralogy of Fallot (F8), Progressive dilatated aortic root and descending aorta (F10), Atrial septum defect (F3a, F14b, F17), muscular or membranous ventricular septum defect (F14a, F14b, F17), Patent ductus arteriosus (F18), Subaortic stenosis (F18), Persistent foramen ovale (F21).
Major malformation of gut	pyloric stenosis (F3a, F22,) anal atresia (F6d), intestinal malrotation (F8)
Diaphragmatic hernia	F17
Gastro-esophageal reflux	Mild (F6b, F6d, F17 only early infancy, F20, F22), Moderate (F6c), Moderate-severe (F5), Severe (F6a), Unspecified (F4, F7 (GE-related apparent life threatening event at age 2 years), F8, F9, F10)
Genitourinary system major anomalies	F14b: Left ectopic kidney
Genitourinary system minor anomalies	Cryptorchidism (F11a, F16a, F17), Abnormal genitalia (F1), Micropenis (F11a), Hypospadias (severe in F11a, mild in F21), Small labia majora (F5, F10), Shawl scrotum (F11a), Bifid scrotum (F11a, F21) Vesicoureteral reflux grade I (F20)
Temperature instability	F6e, F16a, F16b
**Additional major anomalies**	**Affected individual**
Holoprosencephaly (septo-optic dysplasia	F9
Ataxia, dyspraxia and tremor with normal brain MRI	F2
Insomnia	F2
Choanal atresia	F3a, F14b
Cleft palate as part of Pierre Robin sequence	F12
Apnea	F16b
Pneumothorax at birth	
Hemivertebrae	F16a with scoliosis, F21
Proximal underdevelopment of radii with posterior dislocations	F20
Pyloric stenosis	F3a, F22
Anal atresia	F6d
Constipation	F2, F11a, F11b, F16a, F16b
Episodes of marked hypoglycemia	F2

Radioulnar synostosis	F8
Stage 1 melanoma at age 26 years	F11b
Exostoses	F19, F20, F21
**Additional minor anomalies**	**Affected individual**
Oligohydramnios	F21
Arachnoid cyst	F16a
Nystagmus	F11a
Brisk tendon reflexes	F7
Asymmetric skull	F8
Scaphocephaly	F11a, F16a
Sparse scalp hair	F10, F11a, F21
Frontal upsweep	F18
Long face	F11b
Frontal bossing	F11a
Prominent metopic ridge	F21
Upslanting palpebral fissures	F6e, F13
Long palpebral fissures	F21
Astigmatism	F7
Strabismus	F5, F11a, F16a
Underdeveloped malae	F16a
Prominent nasal bridge	F4, F11a, F11b, F22
Broad nasal tip/bulbous nose	F14a, F20, F22
Cutaneous tag right cheek	F14b
Thick upper vermillion	F16a
Crowded teeth	F11b, F18
Underdeveloped teeth enamel	F8
Neonatal tooth	F14a
Velopharyngeal insufficiency	F8
Bifid uvula	F10, F22

Broad uvula	F4
Prominent chin	F18
Posteriorly rotated ears	F11a
Short neck	F6b, F6c, F14a
Broad neck	F6b, F6c
Vertebral cleft	F8
Bilateral fusion of first and second ribs	F20
Thin fingers	F8
Tapering fingers	F12
2-3 finger syndactyly	F4
Camptodactyly	F6d, F21, F22
Broad thumbs	F21
Cone-shaped epiphyses (thumb)	F20
Prominent fetal pads	F17, F18, F21
Absent distal flexion creases fingers	F4
Osteoporosis	F6a, F6b at age 25
Limited knee mobility	F7
Talipes calcaneovalgus	F21
Prominent hallux	F13, F21
Sandal gaps	F7, F9, F12
Renal cyst	F10
Inguinal / umbilical hernia	F11a, F10
Unexplained periodic vomiting/diarrhea	F14a
Reactive airway disease	F2

F: Family number

Supporting Table S4: Individual data on cognitive functioning, behavior and developmental milestones in cohort A (n=29)

Patient ID	F1	F2	F3a	F4	F5	F6a	F6b	F6c	F6d	F6e	F7	F8
Age (year;months)	5	11	3;3	12	1	26	61	60	50	44	5	6
Gender	M	M	F	M	F	F	F	F	F	F	M	M
Cognitive functioning†												
Impaired‡	+	+	+	+	too young	+	+	-	-	+	too young	+
Severity	Mild	Mild	Mild	Mild	too young	Mod/sev	Mod	Normal	Normal	Mild	too young	mild
Developmental milestones												
Affected‡		-	?	+		+				-	+	
Sitting independently§					6	7			10			
First words¶	12	12		36	too young	36					24	
Walking without support†	12-18	12		30	too young			15			12	
Behavioral / psychiatric problems‡‡												
Present	-	+	-	+	-	-	-	+	-	+	+	+
Type§§	none	ADHD¶¶, OCD-B, A, CR, ASD-L	none	ADHD	none (not tested yet)	None	none	anxiety	none	A, OCD-B as a child	ASD-L¶¶. No ADHD.	ADHD
Details	Borderline normal IQ, DD (speech and language). Age 3y: IQ 75. Attends regular school with fairly good level of learning	Mild ID; individual educational program with OT and ST. Diagnosed with dyspraxia. Uses medication for ADHD. Some behavioral side effects of anti-epileptic drugs.	Mild DD: initially no DD; at 3y 3mo, about 6 mo delay	DD (global), LD (primarily in reading). Special education services. Scooting at age 1y, 10-15 words at age 4y	Mild DD	Mod/sev ID	Mod ID; B/G IQ at age 15 y: 39	LD present	LD present. Binet IQ at age 4;7y: 94 (cave type of test: possibly overestimation)	LD; dyslexia. School for children with special needs. Trained as a social and health assistant.	Normal IQ; moderate DD (fine motor and speech). Special education for children with mild LD. Age 3y: Leiter IQ 112, age 5y: WPPSI-III VIQ 89, PIQ 101. Processing speed 69. Insufficient pragmatic language skills. ADOS: expressive language difficulties. Reciprocal social interaction, variability in eye contact. No echolalia, stereotyped behavior or restricted interests. Temper tantrums	Mild ID, severe DD (verbal> motor)

Patient ID	F9	F10	F11a	F11b	F12	F13	F14a	F14b	F15	F16a	F16b	F17	F18
Age (y;mo)	23	4	7;6	46	9	‘Child’	5;11	0	13	17	46	2	6;5
Gender	M	F	M	F	F	F	F	F	M	M	M	M	M
Cognitive functioning†													
Impaired‡	+	+	too young	-	+	+	+	+	+	+	+	too young	+
Severity	Mod/sev	too young	too young	Normal	Mild	Mild	Mild	too young	Mod	Mild	Mild	too young	Mild
Developmental milestones													
Affected‡	+	+	+		-		-			+		-	+
Sitting independently§	5		10		7					12			8,5
First words¶	20	Sev delay	Delayed		12		13			24		15	30
Walking without support†	22	25	14		13		11			18		15	11,5
Behavioral / psychiatric problems‡‡													
Present	+	-	+	+	-		-		+	+	+		+
Type§§	OCD-L, A, CR, ASD-L.	none (not tested yet)	AD(H)D, A, ASD-L¶¶	OCD-B, ASD-L¶¶, social A¶¶, no AD(H)D	none¶¶		no ASD-L		OCD-B, A, ASD-L	AD(H)D, OCD-B, A,CR, AG, SIB, no ASD-L	AD(H)D, A; no OCD-B, A, SIB or ASD-L.		ASD¶¶, A as a small child, rest: none
Details	Mod/sev ID. Is rather talkative. In school for persons with autism.	Severe DD	Age 5y 5mo: WPPSI-IV IQ 107; CELF score 98; OWLS oral composite 92Persistent DD (speech and motor). Roll over at age 10 mo, crawl at age 12 mo. 4-5-word sentences at age 3 y. ADOS: repetitive behavior and interests, handwringing, echolalia, aversion to touch, lining up toys, tiptoe walking. Sensory problems	Completed 2y in college, no reported LD. Sensory problems. Depression in the past.	Mild ID; WISC-III at age 8y; TIQ 65, VIQ 63, PIQ 73.	mild DD	Borderline normal IQ, WPPSI-III IQ age 5y11mo: 73, diagnosis of speech delay at 5y6m	feeding difficulties	Mod ID	mild ID/LD	mild ID	transient mild DD diagnosed at 15 mo, resolved at age 2 y	Mild ID, mild DD (mainly speech, motor planning)

Patient ID	F19	F20	F21	F22
Age (y;mo)	'Child'	2;1	5	22
Gender	M	F	M	M
Cognitive functioning†				
Impaired‡	+	too young	+	+
Severity	not specified	too young	Mild	not specified
Developmental milestones				
Affected‡		+	+	+
Sitting independently§				9
First words¶		22	24	36
Walking without support†		not yet	19	11
Behavioral / psychiatric problems‡‡				
Present		-	-	+
Type§§		no AG, SIB or ESW	None	ADHD, no ESW
Details	DD (severity not specified)	DD (motor/ language)	Mild LD/ID; borderline DD; Griffiths age 4y: 2yrs 10 mo, performance subscales 2y 2mo. Socially well adapted.	DD, ID (severity not specified). Enuresis until teens

n/a: not available, Fxx: family number, M: male, F: female, mo: months, y: year, too young: too young to evaluate (mild) problems in cognitive functioning or severity, LD: learning disability, DD: developmental delay, ID: intellectual disability, OT: occupational therapy, ST: speech therapy, ASD: Autism spectrum disorder, ADOS: autism diagnostic observation scale; B/G: Binet/Goodenough, CELF: clinical evaluation of language fundamentals, Griffiths: Griffiths mental development scales, Leiter: Leiter international performance scales Revised, OWLS: oral and written language scales.

†8 formal test results, others physician reported data.
‡Only scored if sufficient data was available to draw conclusions
§Target age: <12 months
¶Target age <15 months
††Target age <18 months
‡‡Presence of problems in one or more of 8 domains: Attention deficit disorder +/- hyperactivity (AD(H)D; denoted as ADHD if specified by reporter), obsessive-compulsive behavior (OCD-B), anxiety (A), constant roaming (CR), Aggression (AG), self-injurious behavior (SIB), extreme shyness or withdrawal (ESW), autistic-like features (ASD-L). For 5 patients based on formal test results, others physician reported data.
§§'None' signifies: none of the 8 domains affected
¶¶based on formal testing

Supporting Table S5: Available clinical information for cohort B (n=20)

Family	Clinical information and interpretation of pathogenicity
F3b	father of F3a, reportedly 'unaffected'(Ansari et al., 2014), without cardiac anomalies, limb reduction defects, gastrointestinal anomalies or ocular anomalies.
F23	13 year old male (Decipher ID 271431), severe ID, seizures, sleep disturbance, plagiocephaly, strabismus, high palate, microtia, overfolded helix, wide intermamillary distance with inverted nipples, long fingers (CdLS score at least 1). The variant occurred de novo. It was called as a VUS *(uncertain/none)* by the submitters, but based on CADD score of 26.2 and literature it is likely pathogenic.
F24	2 year old female with delayed speech and language development, motor delay, microcephaly, coarctation of aorta, patent ductus arteriosus, left ventricular noncompaction cardiomyopathy, feeding difficulties, and history of intrauterine growth retardation and failure to thrive in infancy (CdLS score at least 3).
F25	F26a: 14 year old girl with mild ID, long philtrum, synophrys, prominent fingertip pads, clinodactyly of the 5th finger, proportionate short stature, abnormal sacrum morphology, gastroesophageal reflux, Crohn disease, and cutis marmorata, (CdLS score at least 7). Her father, F26b, had synophrys and short stature (CdLS score at least 3). Decipher ID 272901, no additional information received upon request.
F26	10 year old male with ID (Decipher ID 275402), delayed speech and language, and speech apraxia. He had drooling, thick eyebrows, deeply set eyes, pointed chin, prominent nose, short philtrum and scoliosis (CdLS score at least 3). This variant was called as a VUS by the submitters, but based on our protein modeling and dynamics studies and CADD score 28.7, it is likely pathogenic.
F27	F27a is a 10 year old female, identified in a cohort of patients with 'a variety of unselected clinical presentations referred for clinical exome sequencing' (Yuan et al., 2019), with global developmental delay, static encephalopathy, autism spectrum disorder, dysmorphic features, microcephaly, medically intractable symptomatic generalized epilepsy, possible Von Willebrand disease, anemia and beta thalassemia trait (CdLS score at least 2). F27b is her affected non-mosaic mother with a 'milder phenotype', including schizophrenia and intellectual disability (CdLS score at least 1). They were not suspected of CdLS prior to testing (updated information).
F28	F28a is a 7-year old girl identified in a cohort of patients with 'a variety of unselected clinical presentations referred for clinical exome sequencing' (Yuan et al., 2019), published also as c.1548delinsTC, p.E518fs (Kruszka et al., 2019). She has developmental delay (motor milestones/speech), middle interhemispheric variant holoprosencephaly (syntelencephaly and heterotopias), microcephaly, staring spells, hypotonia, submucous cleft palate, dysmorphic features including synophrys and hypertelorism, without congenital heart disease, growth delay or limb abnormalities (CdLS score at least

	3). F28b is her affected non-mosaic father with a 'milder phenotype', including slight LD's, synophrys and a submucous cleft palate (CdLS score at least 2). They were not suspected of CdLS prior to testing (updated information).
F29	4 year old child identified in a large mixed cohort of 'undiagnosed patients with suspected genetic conditions' (Lee et al., 2014):. Previously suspected of Williams syndrome or Kabuki syndrome. Microcephaly, language delay, speech delay, affected tooth enamel, large ears, long palpebral fissures, high arched eyebrows, high arched palate, long eyelashes and clinodactyly (CdLS score at least 3). It was called as likely pathogenic by the submitters, which matches the CADD score of 33.0, and our protein modelling supports this.
F30	a 4-generation consanguineous family (Bonora et al., 2015; Deglincerti et al., 2007; Mungan et al., 2003) with updated information from authors.<br>- F30a, F30b and F30c: Homozygous siblings (28 year old male, 26 year old male, and 30 year old female, updated), all three deceased in young adulthood. All three had Mungan Syndrome (OMIM 611376) with chronic intestinal pseudo obstruction (CIPO; severe in F30a and b, requiring parenteral feeding), esophageal a-peristalsis, severe gastroesophageal reflux, long-segment Barrett esophagus, dilated small intestine and mega-duodenum, postnatal weight <-2 SD, long eyelashes, concave nasal ridge, long and smooth philtrum and thin upper lip vermillion. All had normal cognitive functioning and no behavioral or psychiatric problems. None had synophrys, limb defects, diaphragmatic hernia, short 5th finger, or hirsutism. Other features included postnatal growth delay in F30a (155cm) and F30c (148cm), postnatal microcephaly in F30c (-4SD; head circumference missing in F30a and b), hairlessness in F30a, freckles on the face in F30a, F30b, ptosis in F30a, F30b and down-slanting palpebral fissures in F30a, F30b, glaucoma in F30b and F30c, hearing loss due to otosclerosis in F30b, Seizures (for 4 years) in F30b, membranous septal defect in F30a, supravalvular pulmonary stenosis and pulmonary and tricuspid valve regurgitation in F30b, and thick and fibrotic pulmonic valves and trivial pulmonic valve stenosis in F30c, Amenorrhea (possibly secondary to malnutrition) in F30c (CdLS score ≥7, ≥6 and ≥8, respectively).<br>- F30d, e, and f: heterozygous adult sister, father and mother of F30a, b and c (new cases). All had long eyelashes, and gastroesophageal reflux disease. All had normal cognitive functioning, normal prenatal and postnatal head circumference and stature, and none of them had synophrys, concave nasal ridge, long or smooth philtrum, thin upper lip vermillion, limb malformations, small hands or feet, diaphragmatic hernia, cardiac abnormalities or seizures. Other features: mild hearing loss in F30d; arched eyebrows, glaucoma and late-onset Alzheimer disease in F30e; 5th finger clinodactyly in F30f (CdLS score: ≥1 in F30f).<br>- A further 14 confirmed heterozygous family members and all non-tested family members were unavailable for update but included:<br>- Four cousins (of consanguineous heterozygous parents) who died below age 20 and had gastrointestinal problems (possibly) fitting CIPO diagnosis. One of them had also growth retardation, renal hypoplasia, vesico-urethral reflux, ascites, and unspecified granulomatous hepatitis

	- One confirmed heterozygous female with growth retardation<br>- One non-tested cousin with hyperkinetic behavior at age 18 years.<br>The variant in this family was called as pathogenic (CADD score 20.9), and our analyses support pathogenicity at least in homozygotes (Mungan Syndrome with features suggestive of CdLS). Due to lack of clinical data, it remains to be determined whether a full CdLS phenotype can occur, and if this mutation leads to a phenotype in heterozygous form.
F31	Patient with delayed gross motor development, delayed speech and language development, failure to thrive, flexion contracture (unspecified), generalized hypotonia, heart murmur, hyperlordosis, Madelung deformity and oral cleft (score ≥1).
F32	A one month old female with ‘abnormality of the ear’.
F33	Patient with patent ductus arteriosus, atrial septum defect, ventricular septum defect, hearing impairment and coloboma, as well as osteochondrosis dissecans. The microdeletion includes a large distal part of *EXT1*.

F: Family number, ID: intellectual disability

Supporting Table S6: Patients not included in this study with unusual features

Identifier	Description
**Published cases not included in this study**	
**I-III**	Three patients with large *de novo* microdeletions including RAD21 that were part of all previous RAD21-CdLS case series (Deardorff et al., 2012; Pereza et al., 2012; Wuyts et al., 2002) were excluded.<br>- A 12-year old male with an approximately 5.4Mb deletion of 8q23.3q24.12 (published twice) was excluded due to unclear deletion breakpoints (Deardorff et al., 2012; Wuyts et al., 2002). He was originally diagnosed with Langer-Giedeon syndrome (OMIM 150230). CdLS score was ≥11. He had developmental delay and ID (IQ 55-60) and attended a school for slow learners and had combined type epilepsy from age 10. He works as nurse assistant. Features atypical for CdLS included exostoses, telecanthus, kyphosis, barrel chest, short metatarsals, absent pubic hair and T2 weighed hyperintensities on MRI brain.<br>- A 3,5 year old female with arr[hg19] 8q23.3q24.13(116852070-124373809)x1 was excluded due to overlap with multiple other disease related genes (Pereza et al., 2012). She was originally diagnosed with Langer-Giedeon syndrome (OMIM 150230). CdLS score was ≥11. She had mild developmental delay (mainly speech, dyslalia) but cognition was tested normal and there were no behavioral problems. She had feeding difficulties and failure to thrive. Features atypical for CdLS included above average stature and weight, exostoses, delayed bone age, brachyphalangia, brachymetacarpia, mild cone shaped epiphyses, premature adrenarche at age 24 months, bulbous nose, downslant, and sparse and fine scalp hair.<br>- A 7-year old male with arr[hg19] 8q23.3q24.12(117639532-120955012)x1, also excluded due to overlap with multiple other disease related genes (Deardorff et al., 2012). He was diagnosed with CdLS prior to testing, CdLS score was ≥11. He had normal cognitive functioning, and no behavioral problems. Features atypical for CdLS included exostosis, pneumothorax at birth, thoracic vertebral cleft and *coxa vara* with short femoral necks (reported at age 7 years), long 4th metacarpals of the feet, thin temporal scalp hair and hyperpigmentation behind the ears.
**IV**	A 6-year old female with autism and a de novo Phe114Leu variant (Yuen et al., 2015), Moderate ID (Leiter IQ 48 at age 5y 2 months) with some fine motor functioning difficulties but normal gross motor and speech development, without seizures; she had two other variants elsewhere in the genome and it remains uncertain to which extent the phenotype was caused by the *RAD21* variant.
**V**	A 14-year old male with a RAD21 frameshift variant p.(Gln197*) (Kruszka et al., 2019), with holoprosencephaly, severe ID, hypotelorism, synophrys, upturned nose, long philtrum, microcephaly, seizure disorder and no limb anomalies or other major anomalies, or growth delay (CdLS not suspected prior to genetic testing; CdLS score at least 8). He was excluded as it turned out his sister was also severely handicapped, had partially overlapping dysmorphic

	features but had ocular hypertelorism, and did not have holoprosencephaly, and RAD21 status had not been confirmed (updated information). At the present no conclusion can be drawn regarding an association between the *RAD21* variant and the phenotype.
**VI-XI**	Six patients from a four-generation family with eight cases of bilateral sclerocornea with autosomal dominant inheritance pattern, linked to RAD21 missense variant p.(Arg450Cys) through WES on 5 affected males and 1 affected female (Zhang, Chan, et al., 2019), with updated clinical information. All had opaque cornea rims, thinner cornea and shallower anterior chamber depth compared to the unaffected family members. Eye features included cataract in one male and one female. Additional enquiry revealed that none were not suspected of CdLS prior to testing. None had ID, thick eyebrows, synophrys, abnormal nasal ridge or lips, oligodactyly, growth retardation, microcephaly, hirsutism, diaphragmatic hernia, 'metabolic syndromes' or systemic abnormalities other than hypertension. Sclerocornea can be secondary to blepharitis or eye rubbing, but this was not reported in this family. There are some reports mentioning corneal opacities in CdLS due to blepharitis and eye rubbing from the era before causative genes were known, but no earlier report mentions isolated sclerocornea in CdLS (Barr et al., 1971; Ptacek, Opitz, Smith, Gerritsen, & Waisman, 1963; Shi & Levin, 2019).<br>The Arg450Cys variant could not be modeled. The 450 residue is not conserved, but alterations may disrupt the (SX)EXXR(X) consensus motif of one of the two ESP1 separase cleavage sites, involved in cohesin release but might not both be needed for proper release (Hauf, Waizenegger, & Peters, 2001; Lin, Luo, & Yu, 2016; Zhang, Wong, et al., 2019). The variant was reported once in a south Asian individual in GnomAD and has CADD score 24.1. In all, we doubt the relationship between sclerocornea and this variant. However, as the phenotype was replicated in xenopus (Zhang, Wong, et al., 2019), we cautionally consider it a VUS until further evidence for a causative relationship is presented.
**XII-XXII**	All 10 deletions potentially overlapping with RAD21 in Maas et al[30] were too large and/or exact breakpoints were not specified.
**Unpublished deletions/duplications not included in this study**	
**I**	A 3-year old male with a small microdeletion including *RAD21*, *EXT1* and partially overlapping with *SAMD12* (chr 8:117364229-119560288x1, personal communication F. Kaiser). The complete clinical questionnaire revealed mild developmental delay, delayed milestone (first words), microcephaly, scoliosis with hemi-vertebrae, arched eyebrows, long eyelashes, broad or depressed nasal ridge, thin upper lip vermilion, high arched palate, low set or malformed ears, clinodactyly and short 5th finger, bilateral transverse palmar creases, and finger and toe pads and exostoses (CdLS score ≥7).
**II-VI**	4 cases with a microdeletion (Decipher ID's, 305630, 300729, 377614 and 277106), as clinical data were requested but not received
**VII-VIII**	2 small duplications were excluded as they were likely benign. Both included exons 12-14 of *RAD21* as well as genes *EIF3H* and *UTP23*, which likely creates 2 functional copies of RAD21 and one short transcript which will be degraded:<br>- Arr[hg19] 8q23.3-24.11(chr8:117649533-117863721)x3 in a patient with global developmental delay and autism (ClinVar).

	- arr[hg19] 8q23.3q24.11(117621592-117864753)x3, in a patient with schizophrenia (personal communication J. Howe).
**IX-X**	2 small duplications involving *RAD21* were reported to us, but not included in the tables as no phenotype could not reliably attributed (Jennifer Howe, personal communication): arr[hg19] 8q24.11(117836131-117873811)x3 (exons 5-14) and 8q23.3q24.12 (117841222-117899210)x3 (whole gene, found in two unrelated controls.
	All duplications reported on Decipher and all but 1 other duplications on ClinVar were excluded due to substantial overlap with other morbid genes
**Unpublished intragenic variants not included in this study**	
**I-VIII**	Eight cases that were reported in ClinVar as VUS, that were found to either be likely benign, or remain VUS in our analyses, but could potentially turn out to be (likely) pathogenic in the future if more clinical data becomes available:<br>- Missense variant c.418G>A, p.Val140Met in a patient with multiple congenital anomalies, dysmorphic features, FTT/undergrowth, hypotonia, craniofacial anomaly, neurologic and pulmonary problems, and was familial (no data on family members available so it is not known whether these also have a (mild) phenotype). The variant is called as a VUS by the submitters, and has a CADD score of 28.7. Without additional clinical data we must consider this to be a VUS as well.<br>- Missense variant p.(Arg450His) in a 9 year old girl with ID, without dysmorphic features, without growth retardation, skeletal anomalies or congenital anomalies, and was normocephalic. It was called as a VUS by the submitters. Because of the lack of CdLS features, relationship of the ID with the *RAD21* variant was disputed by the submitters, but detailed clinical appraisal was not submitted. GnomAD reports this variant in eight out of 34.000 subjects of Latino descent, CADD score is 22.6. Although the Arg450 is one of the ESP1 separase cleavage sites on RAD21 (see discussion on variant Arg450Cys), this alteration at Arg450 is likely benign.<br>- p.(Leu451Arg) in a patient with a history of neurodevelopmental delay. It is not known whether other CdLS genes were tested. The variant was called by the submitters as a VUS and has high CADD score (31.0), but is an amino acid substitution at a conserved position but does not alter the (SX)EXXR(X) consensus motif (see discussion on variant Arg450Cys). It was also found in 81 subjects in GnomAD, thus is likely benign. At the present, the effect of variants involving residues 450 and 451 remains unclear.<br>- p.(Ala467Val) in a male with multiple congenital anomalies, MR/ID/DD, seizures/epilepsy, dysmorphic features and FTT/undergrowth (score ≥2). The variant was called by the submitters as a VUS, and was found in one GnomAD subject, CADD score 22.3.<br>- p.(Asn511Ser) in a patient with a history of neurodevelopmental delay. The variant was called as a VUS by the submitters, which matches the CADD score of 20.6.

	- p.(Glu526Gln) in a female of European/Asian origin with multiple congenital anomalies, ID/DD, dysmorphic features, Failure to thrive/undergrowth, hypotonia, craniofacial problems, neurologic problems and pulmonary problems. (score at least 2). It was called as a VUS by the submitters, and has a the CADD score of 22.7.<br>- p.(Ser618Gly) in a 13-year old boy and his father. The boy had a history of autism spectrum disorder, ADHD, myopia, sleep issues, anxiety, and epistaxis. The father did not have a history of neurodevelopmental concerns but no other clinical information was available. It was reported as a VUS by the submitters, and CADD score was 20.9. The splice prediction tool of the Alamut software suggests that possibly splicing is affected but this *in silico* prediction could not be confirmed as no RNA was available to check out splicing. As it was still a VUS, we did perform protein analysis on this variant, but it did not cause structural or dynamical changes (see figure 2). However, Ser618 itself is a phosphorylation site (Hegemann et al., 2011; Hornbeck et al., 2015) thus mutations here could be clinically relevant, and not having neurodevelopmental concerns does not exclude a RAD21 phentoype in the father. But for now we have to conclude it remains a VUS due to insufficient clinical information and thus it was excluded from the main cohorts.
**IX**	1 case with a de novo frameshift variant (p.(Val284Leufs*7, Decipher ID 362506), as clinical data was requested but not received
**X-XIII and VIII**	4 cases with missense variants from ClinVar which could be mapped to the crystal structure :<br>- A case with p.Pro355Leu, called as a variant of uncertain significance (VUS). No full clinical information appeared available. Although splice prediction again suggested a possibly affected splicing, this *in silico* prediction could not be confirmed as no RNA was available to check out splicing. Thus, this variant was worthy of modeling. Pro355Leu is a semi-conservative amino acid substitution located in a conserved position. It is located in the non-structured segment of the RAD21-STAG1/2 domain (position is indicated in figure S3), and showed little differences in RMSD trajectory compared to wild-type. Thus, our protein modelling does not suggest pathogenicity of the Pro355Leu variant.<br>- A case with a Gly575Ala variant called as VUS, with a history of neurodevelopmental delay. In retrospect, it was considered by the submitter to be very likely benign due to its occurrence in 6 heterozygotes on GnomAD, and in 2 additional internal cases without phenotypic overlap (personal communication, M. Towne, Ambry). . An *in silico* suggested effect on splining could not be confirmed as no RNA was available to check out splicing. Gly575 is located close to Lys573, a residue described as substrate for ubiquitination (Akimov et al., 2018; Hornbeck et al., 2015) (see figure 2). The variant did not seem to cause any structural alterations. Taken together, it seems unlikely that this variant is pathogenic.

	- A patient with a RAD21 Arg586Gln variant with a history of neurodevelopmental disorder was excluded because upon enquiry it turned out the patient has multiple VUS and a pathogenic truncating mutation in *SCN1A* (OMIM #182389) that was thought to drive the phenotype (personal communication, M. Towne, Ambry). There was no indication for an effect on splicing. In addition, protein modeling showed a clear structural effect. The wild-type Arg586 residue (figure 2) interacts through a salt bridge with *RAD21* residue Glu577, stabilizing the RAD21-SMC1A structure. In the case of variant Arg586Gln, the MD simulation predicts that the interaction with Glu577 is lost, and the Glu577 residue changes its position in the mutant protein by pointing towards the solvent, which adds an additional negative charge to the surface of RAD21-SMC1A. Clinical data were not available, making it impossible to determine whether the RAD21 variant did contribute to the phenotype of the patient. At the present we cannot exclude pathogenicity for the Arg586Gln mutation.<br>- The p.(Ser618Gly) variant (see above).
**XIV-XVIII**	5 cases from ClinVar, each with a variant that has already reported in another patient (Arg450His, Leu451Arg (2 cases), Glu526Gln, and Ser618Gly) were excluded due to unavailability of clinical data. This suggests these are also recurrent variants. The Ser618Gly missense mutation was included in the protein modelling based on another patient in cohort B.

ID: Intellectual disability, DD: developmental disability, VUS: variant of uncertain significance.

Supporting materials D: Literature references with supporting information

Akimov, V., Barrio-Hernandez, I., Hansen, S. V. F., Hallenborg, P., Pedersen, A. K., Bekker-Jensen, D. B., . . . Blagoev, B. (2018). UbiSite approach for comprehensive mapping of lysine and N-terminal ubiquitination sites. *Nat Struct Mol Biol, 25*(7), 631-640. doi:10.1038/s41594-018-0084-y

Ansari, M., Poke, G., Ferry, Q., Williamson, K., Aldridge, R., Meynert, A. M., . . . FitzPatrick, D. R. (2014). Genetic heterogeneity in Cornelia de Lange syndrome (CdLS) and CdLS-like phenotypes with observed and predicted levels of mosaicism. *J Med Genet, 51*(10), 659-668. doi:10.1136/jmedgenet-2014-102573

Barr, A. N., Grabow, J. D., Matthews, C. G., Grosse, F. R., Motl, M. L., & Opitz, J. M. (1971). Neurologic and psychometric findings in the Brachmann-De Lange syndrome. *Neuropadiatrie, 3*(1), 46-66. doi:10.1055/s-0028-1091799

Benkert, P., Biasini, M., & Schwede, T. (2011). Toward the estimation of the absolute quality of individual protein structure models. *Bioinformatics, 27*(3), 343-350. doi:10.1093/bioinformatics/btq662

Bonora, E., Bianco, F., Cordeddu, L., Bamshad, M., Francescatto, L., Dowless, D., . . . De Giorgio, R. (2015). Mutations in RAD21 disrupt regulation of APOB in patients with chronic intestinal pseudo-obstruction. *Gastroenterology, 148*(4), 771-782 e711. doi:10.1053/j.gastro.2014.12.034

ClinVar. Retrieved from https://www.ncbi.nlm.nih.gov/clinvar/?term=RAD21[gene]

Coffee, B., Ikeda, M., Budimirovic, D. B., Hjelm, L. N., Kaufmann, W. E., & Warren, S. T. (2008). Mosaic FMR1 deletion causes fragile X syndrome and can lead to molecular misdiagnosis: a case report and review of the literature. *Am J Med Genet A, 146A*(10), 1358-1367. doi:10.1002/ajmg.a.32261

Deardorff, M. A., Wilde, J. J., Albrecht, M., Dickinson, E., Tennstedt, S., Braunholz, D., . . . Kaiser, F. J. (2012). RAD21 mutations cause a human cohesinopathy. *Am J Hum Genet, 90*(6), 1014-1027. doi:10.1016/j.ajhg.2012.04.019

Decipher. Retrieved from https://decipher.sanger.ac.uk/search?q=rad21#consented-patients/results

Deglincerti, A., De Giorgio, R., Cefle, K., Devoto, M., Pippucci, T., Castegnaro, G., . . . Stanghellini, V. (2007). A novel locus for syndromic chronic idiopathic intestinal pseudo-obstruction maps to chromosome 8q23-q24. *Eur J Hum Genet, 15*(8), 889-897. doi:10.1038/sj.ejhg.5201844

Diebold-Durand, M. L., Lee, H., Ruiz Avila, L. B., Noh, H., Shin, H. C., Im, H., . . . Gruber, S. (2017). Structure of Full-Length SMC and Rearrangements Required for Chromosome Organization. *Mol Cell, 67*(2), 334-347 e335. doi:10.1016/j.molcel.2017.06.010

Gligoris, T. G., Scheinost, J. C., Burmann, F., Petela, N., Chan, K. L., Uluocak, P., . . . Lowe, J. (2014). Closing the cohesin ring: structure and function of its Smc3-kleisin interface. *Science, 346*(6212), 963-967. doi:10.1126/science.1256917

Gudmundsson, S., Anneren, G., Marcos-Alcalde, I., Wilbe, M., Melin, M., Gomez-Puertas, P., & Bondeson, M. L. (2018). A novel RAD21 p.(Gln592del) variant expands the clinical description of Cornelia de Lange syndrome type 4 - Review of the literature. *Eur J Med Genet*. doi:10.1016/j.ejmg.2018.08.007

Hara, K., Zheng, G., Qu, Q., Liu, H., Ouyang, Z., Chen, Z., . . . Yu, H. (2014). Structure of cohesin subcomplex pinpoints direct shugoshin-Wapl antagonism in centromeric cohesion. *Nat Struct Mol Biol, 21*(10), 864-870. doi:10.1038/nsmb.2880

Hauf, S., Waizenegger, I. C., & Peters, J. M. (2001). Cohesin cleavage by separase required for anaphase and cytokinesis in human cells. *Science, 293*(5533), 1320-1323. doi:10.1126/science.1061376

Hegemann, B., Hutchins, J. R., Hudecz, O., Novatchkova, M., Rameseder, J., Sykora, M. M., . . . Peters, J. M. (2011). Systematic phosphorylation analysis of human mitotic protein complexes. *Sci Signal, 4*(198), rs12. doi:10.1126/scisignal.2001993

Hornbeck, P. V., Zhang, B., Murray, B., Kornhauser, J. M., Latham, V., & Skrzypek, E. (2015). PhosphoSitePlus, 2014: mutations, PTMs and recalibrations. *Nucleic Acids Res, 43*(Database issue), D512-520. doi:10.1093/nar/gku1267

Humphrey, W., Dalke, A., & Schulten, K. (1996). VMD: visual molecular dynamics. *J Mol Graph, 14*(1), 33-38, 27-38.

Kline, A. D., Moss, J. F., Selicorni, A., Bisgaard, A. M., Deardorff, M. A., Gillett, P. M., . . . Hennekam, R. C. (2018). Diagnosis and management of Cornelia de Lange syndrome: first international consensus statement. *Nat Rev Genet, 19*(10), 649-666. doi:10.1038/s41576-018-0031-0

Kruszka, P., Berger, S. I., Casa, V., Dekker, M. R., Gaesser, J., Weiss, K., . . . Muenke, M. (2019). Cohesin complex-associated holoprosencephaly. *Brain, 142*(9), 2631-2643. doi:10.1093/brain/awz210

Lee, H., Deignan, J. L., Dorrani, N., Strom, S. P., Kantarci, S., Quintero-Rivera, F., . . . Nelson, S. F. (2014). Clinical exome sequencing for genetic identification of rare Mendelian disorders. *JAMA, 312*(18), 1880-1887. doi:10.1001/jama.2014.14604

Lin, Z., Luo, X., & Yu, H. (2016). Structural basis of cohesin cleavage by separase. *Nature, 532*(7597), 131-134. doi:10.1038/nature17402

Marcos-Alcalde, I., Mendieta-Moreno, J. I., Puisac, B., Gil-Rodriguez, M. C., Hernandez-Marcos, M., Soler-Polo, D., . . . Gomez-Puertas, P. (2017). Two-step ATP-driven opening of cohesin head. *Sci Rep, 7*(1), 3266. doi:10.1038/s41598-017-03118-9

Mungan, Z., Akyuz, F., Bugra, Z., Yonall, O., Ozturk, S., Acar, A., & Cevikbas, U. (2003). Familial visceral myopathy with pseudo-obstruction, megaduodenum, Barrett's esophagus, and cardiac abnormalities. *Am J Gastroenterol, 98*(11), 2556-2560. doi:10.1111/j.1572-0241.2003.08707.x

Pereza, N., Severinski, S., Ostojic, S., Volk, M., Maver, A., Dekanic, K. B., . . . Peterlin, B. (2012). Third case of 8q23.3-q24.13 deletion in a patient with Langer-Giedion syndrome phenotype without TRPS1 gene deletion. *Am J Med Genet A, 158A*(3), 659-663. doi:10.1002/ajmg.a.35201

Ptacek, L. J., Opitz, J. M., Smith, D. W., Gerritsen, T., & Waisman, H. A. (1963). The Cornelia De Lange Syndrome. *J Pediatr, 63*, 1000-1020. doi:10.1016/s0022-3476(63)80234-6

Shi, A., & Levin, A. V. (2019). Ophthalmologic findings in the Cornelia de Lange syndrome. *Ophthalmic Genet, 40*(1), 1-6. doi:10.1080/13816810.2019.1571617

Wuyts, W., Roland, D., Ludecke, H. J., Wauters, J., Foulon, M., Van Hul, W., & Van Maldergem, L. (2002). Multiple exostoses, mental retardation, hypertrichosis, and brain abnormalities in a boy with a de novo 8q24 submicroscopic interstitial deletion. *Am J Med Genet, 113*(4), 326-332. doi:10.1002/ajmg.10845

Yuan, B., Neira, J., Pehlivan, D., Santiago-Sim, T., Song, X., Rosenfeld, J., . . . Liu, P. (2019). Clinical exome sequencing reveals locus heterogeneity and phenotypic variability of cohesinopathies. *Genet Med, 21*(3), 663-675. doi:10.1038/s41436-018-0085-6

Yuen, R. K., Thiruvahindrapuram, B., Merico, D., Walker, S., Tammimies, K., Hoang, N., . . . Scherer, S. W. (2015). Whole-genome sequencing of quartet families with autism spectrum disorder. *Nat Med, 21*(2), 185-191. doi:10.1038/nm.3792

Zhang, B. N., Chan, T. C. Y., Tam, P. O. S., Liu, Y., Pang, C. P., Jhanji, V., . . . Chu, W. K. (2019). A Cohesin Subunit Variant Identified from a Peripheral Sclerocornea Pedigree. *Dis Markers, 2019*, 8781524. doi:10.1155/2019/8781524

Zhang, B. N., Wong, T. C. B., Yip, Y. W. Y., Liu, Z., Wang, C., Wong, J. S. C., . . . Chu, W. K. (2019). A sclerocornea-associated RAD21 variant induces corneal stroma disorganization. *Exp Eye Res, 185*, 107687. doi:10.1016/j.exer.2019.06.001
